# High-Grade Glioma Treatment Response Monitoring Biomarkers: A Position Statement on the Evidence Supporting the Use of Advanced MRI Techniques in the Clinic, and the Latest Bench-to-Bedside Developments. Part 2: Spectroscopy, Chemical Exchange Saturation, Multiparametric Imaging, and Radiomics

**DOI:** 10.3389/fonc.2021.811425

**Published:** 2022-02-28

**Authors:** Thomas C. Booth, Evita C. Wiegers, Esther A. H. Warnert, Kathleen M. Schmainda, Frank Riemer, Ruben E. Nechifor, Vera C. Keil, Gilbert Hangel, Patrícia Figueiredo, Maria Del Mar Álvarez-Torres, Otto M. Henriksen

**Affiliations:** ^1^ School of Biomedical Engineering and Imaging Sciences, King’s College London, St. Thomas’ Hospital, London, United Kingdom; ^2^ Department of Neuroradiology, King’s College Hospital NHS Foundation Trust, London, United Kingdom; ^3^ Department of Radiology, University Medical Center Utrecht, Utrecht, Netherlands; ^4^ Department of Radiology & Nuclear Medicine, Erasmus MC, Rotterdam, Netherlands; ^5^ Department of Biophysics, Medical College of Wisconsin, Milwaukee, WI, United States; ^6^ Mohn Medical Imaging and Visualization Centre (MMIV), Department of Radiology, Haukeland University Hospital, Bergen, Norway; ^7^ Department of Clinical Psychology and Psychotherapy International Institute for the Advanced Studies of Psychotherapy and Applied Mental Health, Babes-Bolyai University, Cluj-Napoca, Romania; ^8^ Department of Radiology and Nuclear Medicine, Amsterdam UMC, location VUmc, Amsterdam, Netherlands; ^9^ Department of Neurosurgery & High-Field MR Centre, Department of Biomedical Imaging and Image-Guided Therapy, Medical University Vienna, Vienna, Austria; ^10^ Department of Bioengineering and Institute for Systems and Robotics - Lisboa, Instituto Superior Técnico, Universidade de Lisboa, Lisbon, Portugal; ^11^ Biomedical Data Science Laboratory, ITACA, Universitat Politècnica de València, Valencia, Spain; ^12^ Department of Clinical Physiology, Nuclear medicine and PET, Copenhagen University Hospital Rigshospitalet, Copenhagen, Denmark

**Keywords:** high-grade glioma, glioblastoma, treatment response, monitoring biomarker, MRI, spectroscopy, CEST, radiomics

## Abstract

**Objective:**

To summarize evidence for use of advanced MRI techniques as monitoring biomarkers in the clinic, and to highlight the latest bench-to-bedside developments.

**Methods:**

The current evidence regarding the potential for monitoring biomarkers was reviewed and individual modalities of metabolism and/or chemical composition imaging discussed. Perfusion, permeability, and microstructure imaging were similarly analyzed in Part 1 of this two-part review article and are valuable reading as background to this article. We appraise the clinic readiness of all the individual modalities and consider methodologies involving machine learning (radiomics) and the combination of MRI approaches (multiparametric imaging).

**Results:**

The biochemical composition of high-grade gliomas is markedly different from healthy brain tissue. Magnetic resonance spectroscopy allows the simultaneous acquisition of an array of metabolic alterations, with choline-based ratios appearing to be consistently discriminatory in treatment response assessment, although challenges remain despite this being a mature technique. Promising directions relate to ultra-high field strengths, 2-hydroxyglutarate analysis, and the use of non-proton nuclei. Labile protons on endogenous proteins can be selectively targeted with chemical exchange saturation transfer to give high resolution images. The body of evidence for clinical application of amide proton transfer imaging has been building for a decade, but more evidence is required to confirm chemical exchange saturation transfer use as a monitoring biomarker. Multiparametric methodologies, including the incorporation of nuclear medicine techniques, combine probes measuring different tumor properties. Although potentially synergistic, the limitations of each individual modality also can be compounded, particularly in the absence of standardization. Machine learning requires large datasets with high-quality annotation; there is currently low-level evidence for monitoring biomarker clinical application.

**Conclusion:**

Advanced MRI techniques show huge promise in treatment response assessment. The clinical readiness analysis highlights that most monitoring biomarkers require standardized international consensus guidelines, with more facilitation regarding technique implementation and reporting in the clinic.

## 1 Introduction

Contemporaneous, accurate, and reliable monitoring biomarkers are required for high-grade glioma treatment response assessment as important challenges limit the use of conventional structural MRI protocols. The current evidence regarding the potential for monitoring biomarkers based on advanced MRI techniques shows that the methodology has developed considerably. Although some techniques have evolved and matured over three decades, several new state-of-the-art methods are poised to contribute to the imaging armamentarium. However, limitations for all techniques remain. High level evidence (level 1 or 2) (1) of clinical diagnostic accuracy typically is lacking. Clinical implementation of standardized tools generally remains challenging, and some recent techniques are in their infancy. Many of these findings were shown following review of the modalities of perfusion, permeability, and microstructure imaging, described in Part 1 (*High-Grade Glioma Treatment Response Monitoring Biomarkers: A Position Statement on the Evidence Supporting the Use of Advanced MRI Techniques in the Clinic, and the Latest Bench-to-Bedside Developments. Part 1: Perfusion and Diffusion Techniques*) of this two-part review article.

The challenges limiting the use of conventional structural MRI protocols as monitoring biomarkers and the need for novel monitoring biomarkers are also described in Part 1. To complete a summary of the evidence for the use of advanced MRI techniques as monitoring biomarkers in the clinic, and to finish highlighting the latest bench-to-bedside developments, we now focus on the individual modalities of metabolism and/or chemical composition imaging. We also appraise the clinic readiness of all the individual modalities. Furthermore, we consider post-processing methodologies involving the combination of MRI approaches (multiparametric imaging) or machine learning (radiomics).

## 2 Materials and Methods

The review method is described fully in Part 1. Briefly, experts in advanced MRI techniques applied to high-grade glioma treatment response assessment, convened through a European framework. The consensus decision was to focus on monitoring biomarkers that can reliably differentiate post-treatment-related effects (PTRE) from true tumor progression during (or before) the point when contrast enhancement on longitudinal relaxation time *T*_1_-weighted MRI images first increases.

Advanced imaging technique analyses were compiled by subject matter experts and incorporated into a manuscript and circulated to the working group members.

To determine clinical diagnostic accuracy, we performed MEDLINE (including PubMed), Embase and Cochrane Register searches for recent systematic reviews and meta-analyses, favoring those which followed Preferred Reporting Items for Systematic Reviews and Meta-Analysis: Diagnostic Test Accuracy (PRISMA-DTA) methodology (2). We also performed searches to analyze individual clinical studies related to each advanced imaging technique since the time of the included systematic review; if a systematic review was published before 2015, we confined our searches to 2015–2021.

## 3 Results

### 3.1 Advanced MRI Techniques

#### 3.1.1 Spectroscopy-Based Techniques

##### 3.1.1.1 Methodology

Proton magnetic resonance spectroscopy (^1^H MRS) is a technique that enables noninvasive characterization of certain biochemicals that are intermediates or end products of cellular metabolism, referred to as metabolites, within tissues based on the chemical shift of molecule resonances in relation to water. The area under a metabolite peak in a magnetic resonance (MR) spectrum is directly proportional to the tissue concentration of this metabolite. The major peaks in the brain include resonances of N-acetyl-aspartate (NAA), choline (Cho), creatine (Cre), and glutamate (Glu), but the total number of quantifiable metabolites depends mainly on the pulse sequence used, sequence parameters (e.g., echo time), and static magnetic field strength (3).

It is well known that spectra acquired from brain tumors are markedly different from spectra acquired from healthy brain tissue (4). An elevated Cho concentration and reduced NAA concentration can often be identified in tumors. A decrease in NAA is often interpreted as a loss or dysfunction of neural tissue, while increased Cho levels are thought to reflect the increased cell membrane turnover in tumors. Additional commonly used markers for tumor proliferation and tumor metabolism include increased lactate, myo-inositol, and lipid levels. In normal brain tissue, lactate is present in a barely MRS-detectable concentration. Elevated lactate levels may be the result of anaerobic glycolysis (i.e., the Warburg effect), necrosis, or ischemia. The exact role of myo-inositol is not fully elucidated, but studies have shown that it may reflect the number of viable glial cells in brain tumors (5). Lipid levels correlate with a breakdown of cell membranes through necrosis and, as such, are a marker for high-grade tumors (6). Because the direct estimation of biochemical concentrations in tumor tissue with *in vivo* MRS remains challenging, clinical and research outputs are normally described as ratios to NAA or Cr.

**Figure 1:**
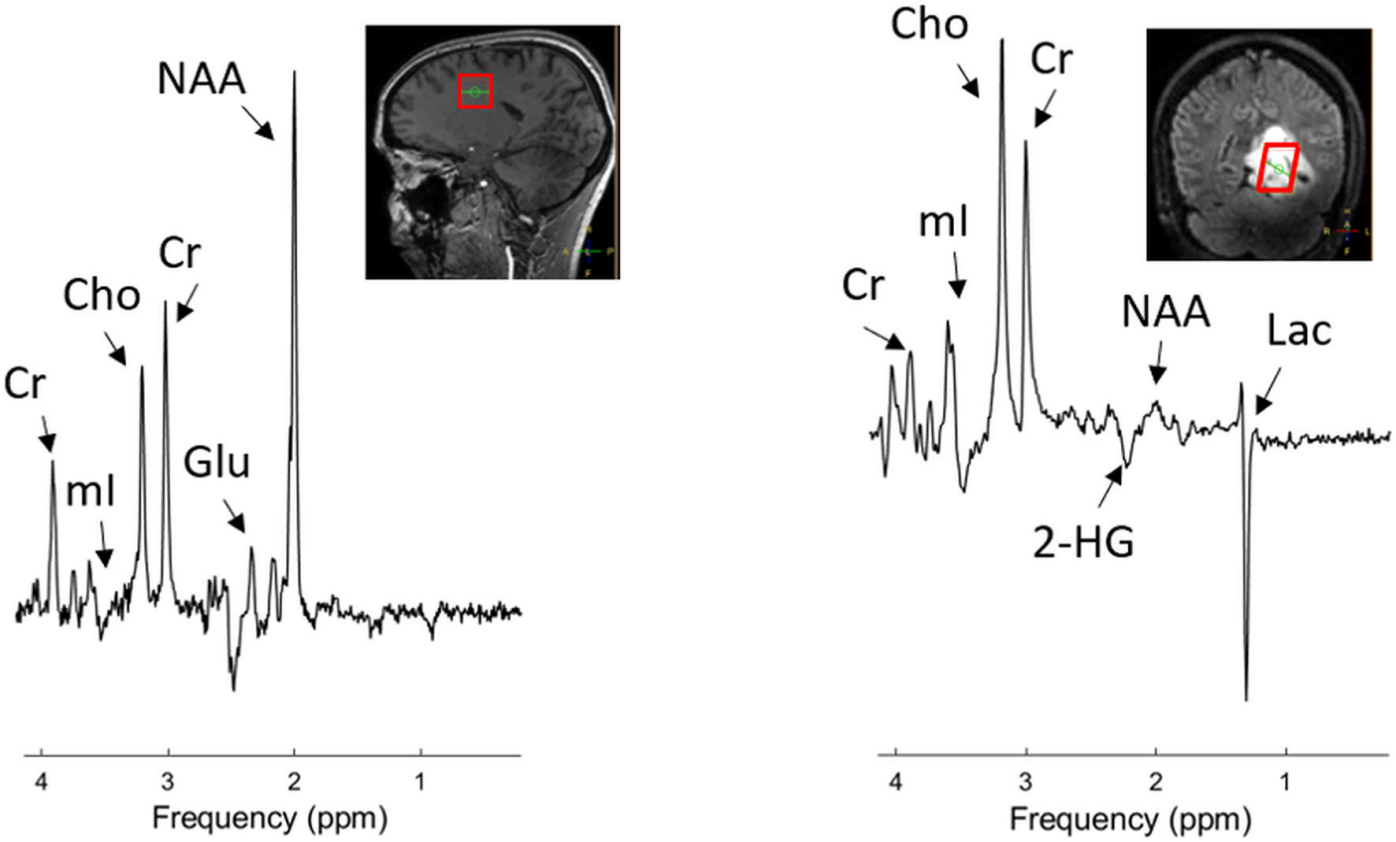
Example of single-voxel ^1^H MRS data acquired in a healthy volunteer (left) and a patient with diffuse astrocytoma with IDH-mutation, WHO grade 2. Data were acquired with a sLASER sequence at 7 T (TE 110 ms, TR 5000 ms) dedicated for detection of 2HG. The location of the MRS voxel is indicated by the red box in the structural images. An elevated Cho and Lac level and reduced NAA level are clearly visible in the tumor. Choline (Cho), Creatine (Cre), N-acetyl-aspartate (NAA), lactate (Lac), myo-Inositol (mI), glutamate (Glu). For illustrative purposes, a low-grade glioma is used.

**Figure 2:**
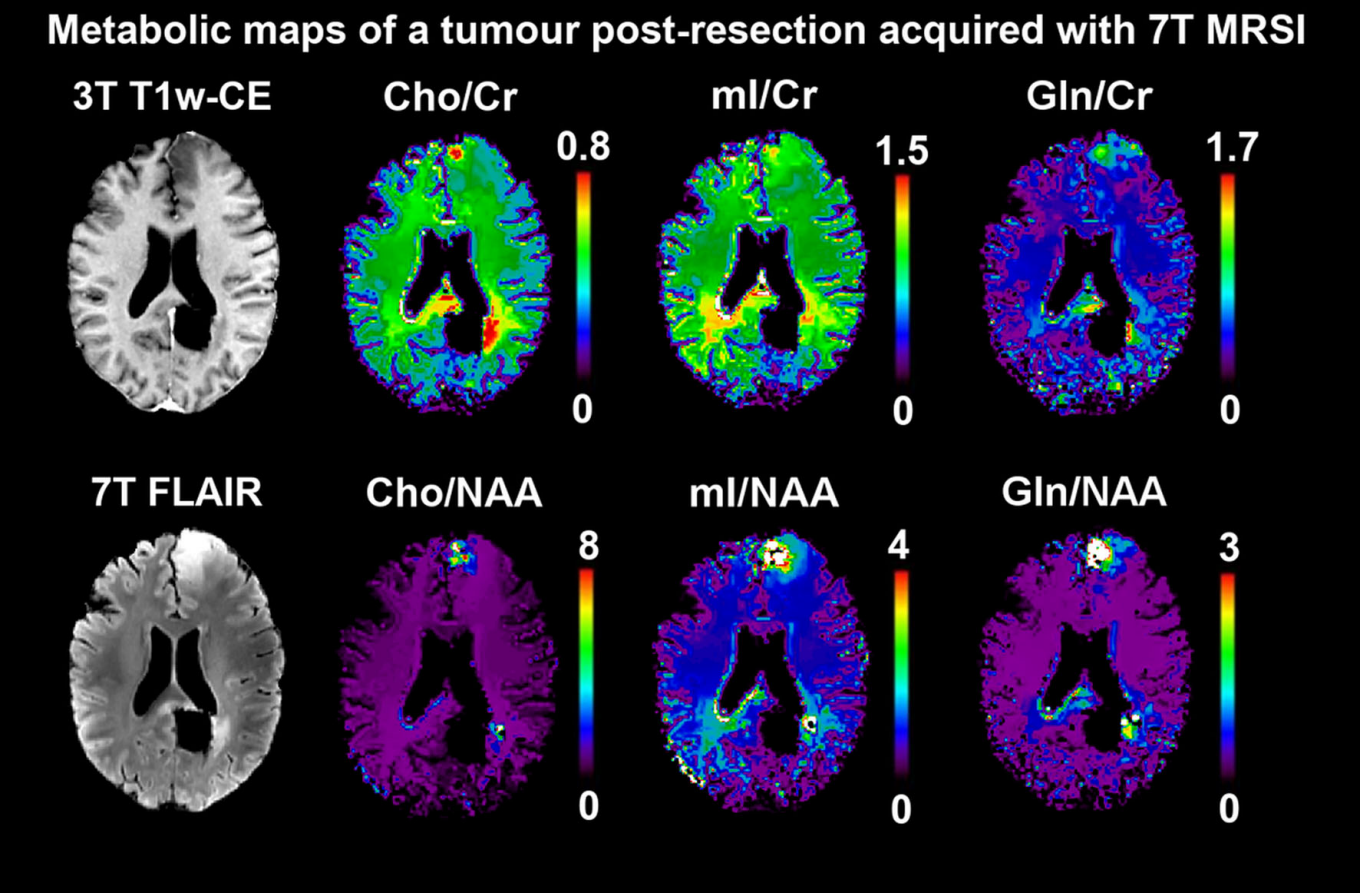
Postsurgical 7 T MRSI scan of a patient with oligodendroglioma, IDH-mutant, 1p/19q deleted, grade 3. Free induction decay-acquisition and patch-based super-resolution, 3.4 × 3.4 × 8 mm³ nominal resolution (7). The ratios of three metabolites (Cho, mI, Gln) to Cr and NAA as common references are mapped. Both in a left frontal second focus as well as around the primary focus resection cavity posterior to the splenium, increased ratios for all six are clearly discernible and relate to morphological findings. Specifically, for the Gln ratios, changes between normal-appearing brain tissues and suspected neoplastic growth are in the range of over a magnitude, making it an attractive potential biomarker, but will require ultra-high-field systems for quantification. Similar techniques could be applied for the spatial identification of neoplastic activity during therapy. Choline (Cho), Creatine (Cr), N-acetyl-aspartate (NAA), myo-Inositol (mI), glutamine (Gln).

^1^H MRS data can be acquired either as single voxel spectroscopy (SVS, **Figure 1**) or from multiple voxels by spectroscopic imaging [2D or 3D magnetic resonance spectroscopic imaging (MRSI), **Figure 2**]. SVS is easy to implement and less time consuming than MRSI. However, the acquisition of a single, rather large, voxel may result in either incomplete sampling of the tumor or the inclusion of peritumoral regions in the sample, which may confound the analysis of heterogeneous tumor tissue.

Recently, the MRS community has tried to move forward and reach a standard consensus regarding MRS methodology developments of the last decade (8–11), but reproducibility studies have not been able to adequately reflect these recent discussions. In particular, multicenter reproducibility studies remain limited to only a few MRS applications (12, 13).

##### 3.1.1.2 Evidence From Clinical Studies

The utility of MRS to distinguish recurrent tumors from radiation necrosis has been evaluated in two meta-analyses to date (**Table 1**). The first meta-analysis (23), comprising 13 studies, evaluated the diagnostic effectiveness of 1H MRS (both SVS and MRSI) in differentiating recurrent tumor from radiation necrosis. This study showed that the Cho/Cr and Cho/NAA ratios are higher in tumor recurrence compared with radiation necrosis (pooled difference: 0.77, 95% CI = 0.57 to 0.98 for Cho/Cr; pooled difference: 1.02, 95% CI = 0.03 to 2.0 for Cho/NAA). In another meta-analysis of 18 studies (20), the pooled sensitivity and specificity of Cho/Cr and Cho/NAA in discriminating recurrent glioma and radiation necrosis are reported to be between 80–90%. Therefore, the authors recommended using MRS as an add-on to the structural MRI.

**Table 1:** Meta-analyses of advanced MRI treatment response monitoring biomarkers. Post-processing methodology meta-analyses are not included here, and are described in the relevant sections below.

Paper	Quality assessment	Period	Modality	Studies/patient (n/n)	Sample size range(n-n)	Prospective Studies (n/n)	Progression compared to:	Pooled measure (n studies)	Sensitivity	Specificity
Yu et al. (14)	Q2	2012-2017	DWI/ADC	6/214	20-68	1/6	PSP	ADC mean (3) 5^th^ centile ADC (2) relative ADC (1)	95 (89-98)	83 (72-91)
Zhang et al. (15)	Q2	2007-2014	DWI/ADC	9/284	20-210	1/9	RN	ADC ratio (7) ADC value (2)	82 (75-91)	84 (76-91)
Okuchi et al. (16)	Q2	2011-2015	DCE	9/298	14-79	3/9	PTRE	All	88 (74-95)	86 (78-91)
							–	K^trans^ (6)	75 (63-84)	79 (68-87)
							–	Toft/Extended Toft (6)	77 (65-86)	85 (75-92)
							–	Model independent (4)	94 (86-98)	85 (74-93)
Patel et al. (17)	Q2	2009-2015	DSC	15/897	9-169	7/28	PTRE	DSC best parameter	90 (85-94)	88 (83-92)
							–	DSC max nCBV (5)	93 (86-98)	76 (66-85)
							–	DSC mean nCBV (8)	88 (81-94)	88 (78-95)
		2011-2015	DCE	7/581	18-57	2/7	–	best parameter	89 (78-96)	85 (77-91)
Wan et al. (18)	Q2	2011-2016	DSC	11/116	20-68	1/11	PsP	nCBV	88 (84-92)	77 (89-84)
Deng et al. (19)	Q	1992-2013	DSC	7/174	10-57	0/18	No progression	rCBV (6)	88 (82-93)	85 (75-92)
Zhang et al. (20)	Q2		MRS	12/262	8-40	1/12	RN	Cho/Cr	83 (77-89)	83 (874-90)
				9/213	13-38	1/12	–	Cho/NAA	88 (81-93)	86 (76-93)
van Dijken et al. (21)	Q2	2009-2014	DSC	18/708	7-90	8/18	PTRE	Best parameter	87 (82-91)	87 (77-91)
		2011-2013	DCE	5/207	13-79	2/5	–	–	92 (73-98)	85 (76-92)
		2006-2014	MRS	9/203	12-40	4/9	–	–	91 (79-97)	95 (65-99)
		2010-2014	ADC	7/204	16-51	4/7	–	–	71 (60-80)	87 (77-93)
		2008-2013	Structural MRI	5/166	7-93	2/8	–	–	68 (51-81)	77 (45-93)
Wang et al. (22)	Q2	2009-2019	DSC	20/939	16-98	5/20	PTRE	nCBV (17) max rCBV (3)	83 (79-86)	83 (78-87)
		2013-2019	DCE	4/250	40-98	1/4	–	K^trans^	73 (66-80)	80 (69-88)
		2013-2018	ASL	3/160	29-69	0/3	–	nCBF	79 (69-87)	78 (67-87)

RN, radiation necrosis; PSP, pseudoprogression; PTRE, post-treatment related effects; Q, QUADAS (Quality Assessment of Diagnostic Accuracy Studies) tool; Q2, QUADAS-2 tool.

In a meta-analysis comparing the diagnostic accuracy of anatomical and advanced MRI [i.e., apparent diffusion coefficient (ADC), dynamic susceptibility contrast-enhanced (DSC), dynamic contrast enhanced (DCE), arterial spin labeling (ASL), and ^1^H MRS (SVS and MRSI)] for treatment response assessment in high-grade gliomas, ^1^H MRS was found to have the highest diagnostic accuracy, with a sensitivity of 91% and specificity of 95%, among all the advanced MRI techniques (21). Various metabolite ratios were used in the MRS studies included in this meta-analysis, but in the majority of the studies Cho/Cr turned out to be the best predictor to differentiate true tumor progression from PTRE. It is noteworthy that in all of the studies above, no explicit description was given as to which part of the tumor (e.g. contrast-enhancing, *T*_2_-weighted hyperintense, or necrotic component) was assessed.

The utility of MRS to differentiate pseudoprogression from tumor recurrence is less well studied, but a few studies show its effectiveness. The potential of 3D MRSI was illustrated in a recent study using 3D echo planar spectroscopic imaging in glioblastoma patients (24). Here, Cho/NAA and Cho/Cr maps were co-registered to anatomical images and mapped on different regions of the neoplasm. Higher Cho/NAA and Cho/Cr ratios specifically in the contrast-enhancing part of the tumor were found in patients with tumor progression compared with patients with pseudoprogression, with a discriminatory accuracy of 94%. Similar results were found in another MRSI study where a threshold of Cho/NAA ≥ 1.3 in the contrast-enhancing part of the tumor was proposed to determine tumor recurrence (25).

##### 3.1.1.3 Strengths and Weaknesses

The main strength of MRS techniques for *in vivo* tumor assessment is the ability to acquire an array of metabolic alterations in one measurement and the flexibility to optimize methods for specific targets of interest. The main limitations of SVS and, to a lesser extent MRSI, are the relatively large voxel size and poor spatial coverage (3). This can lead to partial volume effects between active tumor, treatment-induced changes, and necrosis, as well as the omission of potentially neoplastic tissues. Furthermore, scan time is typically long, artifacts from transcranial lipids or susceptibility differences reduce spectral fitting reliability, and extensive offline processing is usually required. Advanced acquisition techniques can address most of these limitations but require expert operators and tools, and have led to a multitude of published methodologies lacking direct comparability. Therefore, MRS often is not included in routine clinical protocols. Recent initiatives for consensus on MRS methodology and applications are expected to lead to a more “even playing field” and standardized approaches that will make future studies more comparable (9, 26, 27).

##### 3.1.1.4 Future Developments

In most studies on PTRE, only the most prominent MRS peaks (i.e., NAA, Cho, and Cr) have been evaluated as these produce the most signal and are least affected by J-coupling under long echo times. The use of ultra-high field ^1^H MRS (i.e., ≥ 7 Tesla [T]) results in an increased signal-to-noise ratio and an improved ability to separate overlapping peaks (28). Applying 3D MRSI may overcome the barrier of incomplete tumor sampling in SVS, and this has motivated the development of fast and high-resolution spectroscopic imaging sequences (29). With this, additional markers for tumor proliferation and tumor metabolism, including glycine (Gly), Glu, and glutamine (Gln), can be evaluated unambiguously (30). Recently, it was shown in preoperative patients that metabolic differences between tumor regions and peritumoral tissue, beyond decreased NAA levels and elevated Cho levels, could be detected at 7 T (31). For example, high levels of Gln and Gly (which are difficult to separate from Glu and myo-inositol, respectively, at lower fields) were found within the tumor region, which may reflect cancer cell proliferation in the case of Gly and malignant metabolic changes for Gln. Whether these high-resolution 3D metabolite maps could aid in identifying PTRE is yet to be determined. There are high expectations for the application of machine-learning-driven classification of neoplastic tissues that could help to reach this goal (32).

A specific metabolite of interest is 2-hydroxyglutarate (2HG). 2HG is an oncometabolite, produced in glial tumor cells bearing an isocitrate dehydrogenase (IDH) gene mutation, either IDH1 or IDH2. The discovery that 2HG can be detected *in vivo* by dedicated MRS sequences has led to several successful studies showing the ability to determine IDH status noninvasively by MRS (33). Additionally, a potential role for 2HG MRS has been proposed in treatment response imaging. In patients with IDH-mutant tumors, 2HG levels decrease following adjuvant radiation and chemotherapy (34, 35) and increase in the case of tumor progression (35). Furthermore, monitoring 2HG levels could be of specific interest in evaluating the effects of IDH-inhibitors, as was shown in a phase 1 clinical trial (36).

**Figure 3:**
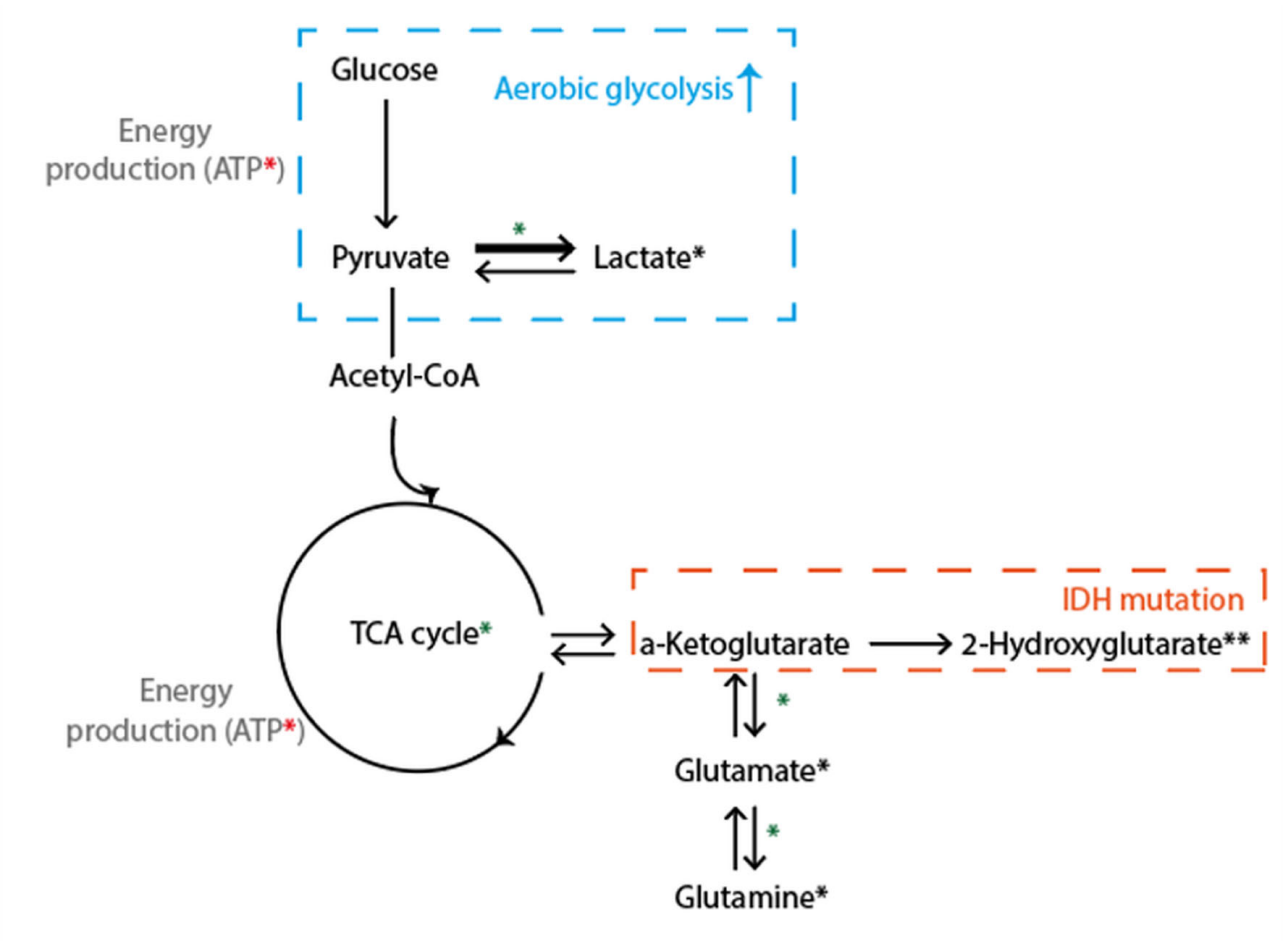
Simplified schematic illustration of key metabolic pathways probed with spectroscopy. Glu (from brain-feeding arteries) is taken up by tumor cells and converted into pyruvate, which enters the tricarboxylic acid cycle and undergoes oxidative metabolism, for the production of energy (ATP). ^1^H MRS visible metabolites are marked with a black *, where ** denotes that a dedicated MRS sequence is needed. Green *: includes pathways visible with ^13^C or DMI. Red *: visible with ^31^P MRS. Adenosine triphosphate (ATP), Glucose (Glu).

Although ^1^H MRS gives insight into steady-state metabolite concentrations, protons are not the only nuclei with resonances of interest. Techniques using other nuclei can be used such as ^31^P MRS and MRSI (37), deuterium metabolic imaging (DMI), and (hyperpolarized) ^13^C MRS and MRSI, which enable the evaluation of tissue metabolism *in vivo* (**Figure 3**). For example, ^31^P-MRSI has been applied to the imaging of inter- and intracellular pH in gliomas, finding increased pH values both at 7 T (37) and 9.4 T (38) in proof-of-concept studies.

These techniques can be used to detect different sets of molecules important to tumor metabolism, such as glucose or ATP, and there is the potential for deriving enzyme activity or acidity. Currently, these techniques are used mainly in a research setting but are potentially promising for distinguishing PTRE, as metabolic reprogramming is one the hallmarks of cancer. For example, it was shown that DMI can be used to visualize tumor tissue metabolism beyond glucose uptake and, thus, map the Warburg effect, which is typically only seen in active tumor cells (39). As such, DMI may be potentially useful to differentiate between treatment-induced necrosis and tumor progression.

#### 3.1.2 Chemical Exchange Saturation Transfer

##### 3.1.2.1 Methodology

**Figure 4:**
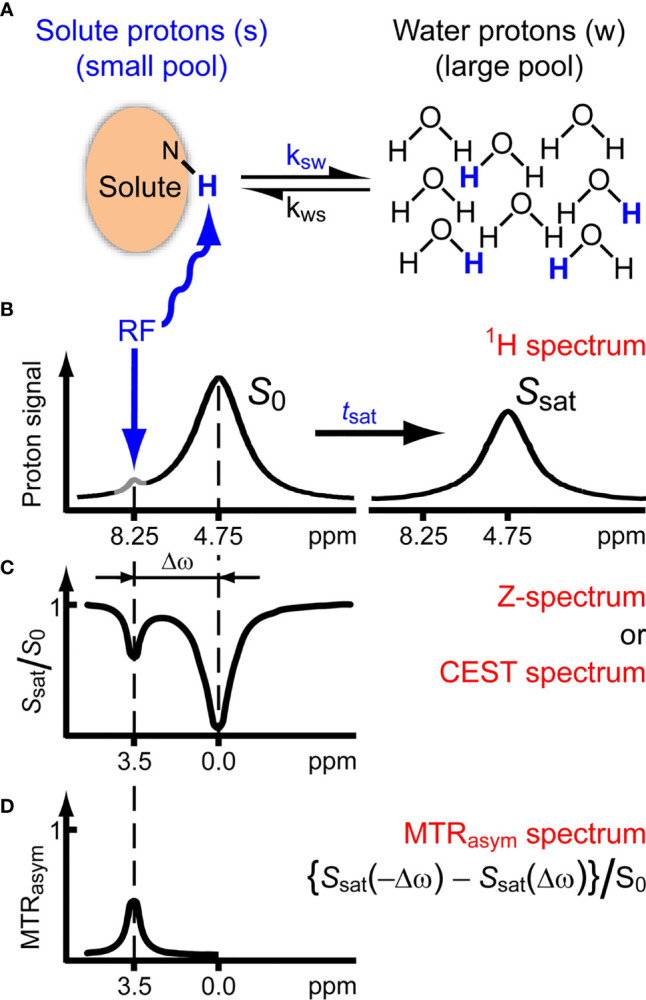
Chemical exchange saturation transfer. **(A, B)** Solute protons (blue) are saturated at their specific resonance frequency in the proton spectrum (here 8.25 ppm for amide protons). This saturation is transferred to water (4.75 ppm) with exchange rate ksw and non-saturated protons (black) return. After a saturation period (t_sat_), this effect becomes visible on the water signal (B, right). **(C)** The Z-spectrum, showing normalized water saturation (S_sat_/S_0_) as a function of irradiation frequency. When irradiating the water protons at 4.75 ppm, the signal disappears due to direct (water) saturation. This frequency is assigned to 0 ppm in Z-spectra. At short saturation times, only this direct saturation is apparent. At longer tsat, the CEST effect becomes visible at the frequency of the low-concentration exchangeable solute protons, now visible at 8.25 – 4.75 = 3.5 ppm in the Z-spectrum. **(D)** Result of MTR_asym_ analysis of the Z-spectrum with respect to the water frequency to remove the effect of direct saturation. Image adapted with permission from (42).

Chemical exchange saturation transfer (CEST) MRI is a technique in which labile protons on endogenous proteins can be selectively targeted to generate contrast (40). In a typical CEST examination in patient studies at 3 T, B_1_ saturation pulses are used with a range of off-resonance frequencies centered around on-resonance B_1_ saturation pulses to generate a Z-spectrum. Labile protons that are bound to mobile proteins are hereby saturated and will lead to saturation of the free water pool when exchanging with the free water protons, depending on their abundance and exchange rate. Endogenous CEST effects that can be targeted include saturation transfer of protons in amide (3.5 ppm), amine (3 ppm), total creatine (Cre) (2 ppm), and hydroxyl (0.9 ppm) bonds. Additional effects of application of off-resonance saturation pulses that will be present within Z-spectra include broad magnetization transfer (MT) effects in semisolid macromolecules, relayed nuclear Overhauser enhancement (NOE) in mobile macromolecules (−1 to −4 ppm) (41), and direct saturation of free water protons (i.e. spillover effect) (42). Note that, in particular at 3 T due to broad spectral linewidths, these effects are either close to or even overlapping with the endogenous CEST effects that are often the target of CEST studies. Several approaches exist to best isolate all of the above effects, such that the CEST effect of interest can be measured. For instance, increasing main magnetic field strength, e.g. using 7 T instead of 3 T systems, aids in separation of all of these effects because it leads to decreased spectral linewidths of the individual effects. Optimizing duration and power of B_1_ saturation pulses can be used to sensitize CEST experiments to protons exchanging with different rates. Analysis approaches include magnetization transfer ratio asymmetry (MTR_asym_) (43), in which signals with off-resonance frequencies with matching positive and negative shift around 0 ppm are subtracted from one another (**Figure 4**), and multiple pool fitting approaches of the Z-spectrum which are used to explicitly isolate individual contributions, such as the NOE, spillover and broad magnetization transfer effects (41, 44). Additionally, a range of methodologies accounts for changes in parameters that will affect the CEST contrast generated. These include additional acquisitions and/or analysis to correct for inhomogeneities in the main magnetic (B_0_) (45) and saturation (B_1_) (46) field, or a change in the *T*_1_ (47).

A full overview of CEST MRI acquisition and analysis approaches is beyond the scope of the current review and has been given previously (40). However, in using CEST MRI for brain tumor imaging some confounding factors do require explicit attention. For example, the *T*_1_ relaxation time of the free water pool and the broad MT effect both directly affect the measured signal in CEST studies. In brain tumors, the *T*_1_ relaxation time is often found to be increased compared to healthy white matter, which is generally attributed to increased tissue water content (48), while changes in macromolecular background in tumor tissue are thought to be the cause for commonly found decreases in MT in brain tumors (49–51). Additionally, B_1_ saturation powers mostly used in CEST brain tumor studies are relatively low (< 2 µT), giving rise to strong NOE effects (41). However, NOE is known to change in brain tumors as well (52). The above highlights the difficulty of isolating the individual components contributing to CEST contrast and that care should be taken when changes in CEST contrasts are attributed to underlying physiological processes. This is an important aspect to keep in mind when reviewing the latest research in applications of CEST MRI to find biomarkers of treatment response in high-grade glioma.

Currently, imaging guidelines are not available (although in preparation). Some technical validation has been performed in healthy subjects in 7 T systems (53).

##### 3.1.2.2 Evidence From Clinical Studies

**Figure 5:**
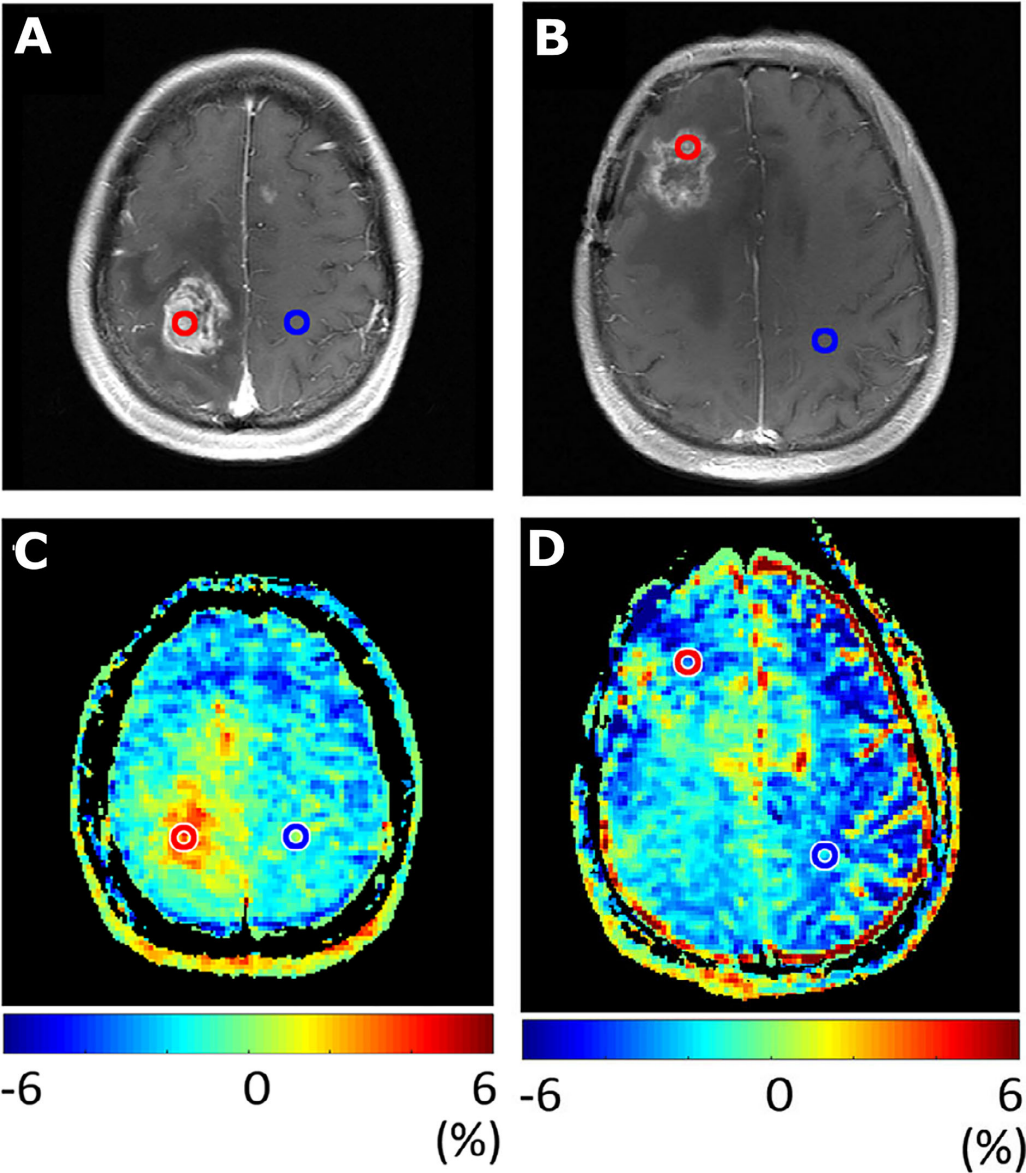
Images illustrating APT-weighted imaging (MTR_asym_ at 3.5 ppm) in two patients after treatment with radiotherapy. Contrast enhancement on *T*_1_-weighted images was seen in patient 1, 62 months after radiotherapy treatment and resection for grade 2 astrocytoma **(A)**. The additional increased MTR_asym_ in the same patient **(C)** illustrates tumor recurrence, which was confirmed as grade 4 glioblastoma after repeat surgery. Contrast enhancement on *T*_1_-weighted images was seen in patient 2, 14 months after chemoradiotherapy of grade 3 astrocytoma, with regional anaplastic oligodendroglioma **(B)**. The additional MTR_asym_ in patient 2 **(D)** illustrates low values, indicating treatment effect, which was confirmed as radiation necrosis with histopathology after repeat surgery. Image adapted with permission from (58).

Amide proton transfer (APT)-weighted CEST is the most investigated CEST technique to derive biomarkers of treatment response. In 2011, it was first shown in preclinical models that the APT-weighted signal of lesions immediately decreases when radiation necrosis occurs (in five animals) (54) or after treatment with temozolomide (five controls, six treated) (55). Increased APT-weighted signal within the lesion after treatment was thought to be indicative of increased cell proliferation in tumor progression, a hypothesis supported by a positive correlation between APT-weighted CEST and Ki67, an immunohistochemical marker of cell proliferation. This correlation has since been reproduced in human gliomas (56) and has led to the first results of increased APT-weighted CEST contrast after treatment to be associated with tumor progression rather than PTRE. However, the application of CEST MRI to differentiate tumor progression from PTRE is a relatively recent development, which has led to only a handful of clinical studies on this topic (see **Table S1**). Two research groups (57, 58) have found that in small cohorts of patients diagnosed with glioblastoma and scanned after chemoradiotherapy or radiotherapy alone, APT-weighted CEST improved differentiation of tumor response from PTRE compared with conventional imaging alone (with a combination of perfusion-weighted and APT-weighted CEST giving the best differentiation). An example of this is presented in **Figure 5**. One of these research groups showed that in even smaller cohorts, APT-weighted CEST outperformed ^1^H MRS (59) and methionine positron emission tomography (PET) (60) at determining tumor progression. Retrospectively comparing APT-weighted CEST with diffusion and perfusion MRI biomarkers also indicated the added value of CEST to elucidate tumor progression in 36 glioblastoma patients treated with chemoradiotherapy or radiotherapy in a recent work (61). In another recent study where APT-weighted CEST was obtained in 32 patients within three months after treatment, increased APT-weighted CEST was seen in tumor progression with radiological confirmation after six months of follow-up (62). Moreover, in a previous, prospective study (50) 19 glioblastoma patients were systematically scanned before, during, and after chemoradiotherapy and an increase in APT-weighted CEST was shown to differentiate progressors from non-progressors as early as two weeks into treatment.

Although the above-referenced studies illustrate clinical findings of elevated APT-weighted CEST at 3 T by several research groups, recent work (63) did not find elevated APT-weighted CEST to be correlated to tumor progression in 12 glioblastoma patients scanned at 7 T. However, when these authors used a combination of image acquisition and analysis aimed at isolating the APT signal from the upfield relayed-nuclear Overhauser enhancement effects, they found that changes in the latter were able to differentiate tumor progression from PTRE. In line with this are the results of prospective studies (64, 65), where patients were scanned with CEST MRI at 7 T before treatment and APT was isolated from NOE effects. This showed that CEST contrasts before treatment are significantly correlated to overall and progression free survival (i.e., a prognostic biomarker). Taken together, these ultra-high field studies highlight the potential of CEST MRI to be used as a prognostic and monitoring biomarker candidate for treatment response assessment, although the different contrasts used indicate yet again that, although CEST contrasts can certainly differentiate active tumor tissue from PTRE, the exact mechanisms causing these contrasts remain to be elucidated.

Other studies optimize CEST image acquisition to be pH-weighted by including (66) or focusing on (67) amine proton exchange, which is thought to be more sensitive to pH changes than cell proliferation. Preclinical work (68) has shown that pH-weighted CEST contrast increases when intracellular pH decreases (i.e., becomes more acidic) in glioblastoma due to chemotherapy. Furthermore, clinical proof-of-concept of using pH-weighted CEST to assess treatment response has been demonstrated in patients after anti-angiogenic treatment (69) and patients treated with combined chemoradiotherapy (70).

##### 3.1.2.3 Strengths and Weaknesses

A strength of CEST MRI for clinical diagnostics in tumor imaging is that those contrasts most explored for tumor imaging arise from endogenous markers and, therefore, no contrast agents are required. Additionally, the process of exchange inherently increases the signal-to-noise ratio of CEST imaging compared with MRS, which allows for a smaller voxel size to be used to probe heterogeneous tissues/pathologies, such as tumors. With these strengths, the potential of CEST MRI to improve differentiation of tumor progression from PTRE is clear. However, weaknesses include the multitude of options to acquire and analyze CEST MRI data, the variation in the timing of CEST MRI included during treatment, the retrospective nature of some of the current clinical studies investigating APT-weighted CEST for tumor treatment response, and the small number of patients in the above-referenced studies. These weaknesses currently prevent a definitive summary of this imaging technique for treatment response assessment, in terms of indications for when to measure and which threshold values to use to separate tumor progression from PTRE.

##### 3.1.2.4 Future Developments

To develop the application of CEST MRI for differentiation of treatment effects and tumor progression, a consensus from all relevant stakeholders regarding image acquisition and analysis is required to enable multicenter and multi-vendor trials. This is an area of active research, where a working group of international CEST experts is working toward an open source consensus CEST acquisition and analysis protocol (71).

#### 3.1.3 Emerging MRI Techniques

There are several emerging techniques that may be shown to be monitoring biomarkers in future proof-of-concept studies. Here, we focus on some studies where proof-of-concept has already been shown.

##### 3.1.3.1 Vascular Architecture Mapping and Oxygenation Imaging

Vessel caliber imaging, or vessel architecture mapping, is based on the fact that when a contrast agent passes through the vasculature and perturbs the local magnetic field, MRI signal from a gradient echo readout is sensitive to large arteries and capillaries, while with a spin echo readout signal is mostly sensitive to capillaries (72). Vessel architecture imaging hereby refers to the modelling framework that aims to assess subvoxel microvascular parameters, such as vessel density and vessel diameter, where vasculature with diameters < 200 µm are targeted (73). This imaging approach is included in recent “tumor microenvironment mapping,” which combines vessel architecture imaging with oxygen metabolism imaging, i.e., measurement of the oxygen extraction fraction with quantitative blood oxygenation level dependent imaging. One study allowed five different tissue types within tumors to be identified (necrosis, hypoxia with/without neovascularization, oxidative phosphorylation, and glycolysis) (74). In 21 tumors scanned pre- and post-treatment, a change in the presence of these five metabolic profiles demonstrated recurrent glioblastoma. Although these results are still very preliminary, this proof-of-concept work shows the potential of this emerging technique to become a future monitoring biomarker.

##### 3.1.3.2 Non-Proton MRI Techniques

Sodium (^23^Na) imaging has established itself in MRI research due to the diverse role of sodium ions in tissue homeostasis (75). Unlike other non-proton techniques such as ^31^P and ^13^C, the ^23^Na signal does not yield a metabolite spectrum, but only a single resonance in most environments such as human tissue (76). Therefore, imaging (as opposed to spectroscopy) is almost exclusively performed for ^23^Na.

Although ^23^Na MRI has been performed successfully in brain cancers since the late 1980s (77), more recent publications have shown its benefit in predicting IDH mutation status and tumor progression (78). Sodium concentration mapping has been performed in recurrent glioblastoma after radiotherapy (79) and also chemoradiotherapy (80). The authors of the former case report showed that the ^23^Na images provided similar information as those contained in [^18^F]fluoro-ethyl-tyrosine (FET) PET images and postulate that ^23^Na images may therefore be able to provide a substitute for PET in MRI-only examination settings (79). Similarly, the authors of the second study noted that the ^23^Na images were sensitive to “real-time” changes in treatment volume that could be used to alter the course of treatment early on (80). Most recently, a study investigated whether whole tumor (excluding necrosis) measured immediately after chemotherapy with a follow-up 6 weeks later could predict stable or progressive disease, but did not find any significant correlations either with treatment response or overall survival (81). As with the other emerging techniques, ^23^Na imaging is best considered as a proof-of-concept technique that may prove to be a future monitoring biomarker.

### 3.2 Advanced Handling of MRI Data

#### 3.2.1 Multiparametric Imaging

##### 3.2.1.1 Multiparametric Advanced MRI

The combination of multiple modalities may be of value for tissue characterization and help differentiate tumor from PTRE by providing complementary information of tumor biology and thus overcome limitations of individual techniques.

##### 3.2.1.2 Evidence From Clinical Studies

A meta-analysis (82) of seven studies of multiparametric MRI (at least two of the following advanced MRI techniques: diffusion tensor imaging (DTI), diffusion-weighted imaging (DWI), DSC, DCE, ASL, and MRS) in patients with suspected pseudoprogression showed a pooled sensitivity and specificity of 84% and 95%, respectively, but the authors noted that the accuracy of multiparametric imaging was not different from that of monoparametric imaging determined in a meta-analysis of individual techniques (21). **Table S2** shows results of studies reporting separate and combined diagnostic performance of ≥ 2 parameters (e.g., PET, DWI, DSC, or MRS). The studies generally showed improved diagnostic accuracy when combining modalities, although the added value may be marginal when compared with the best performing single modality. Combined sensitivity and specificity may even be lower when compared with the single modality that has the highest sensitivity or specificity.

##### 3.2.1.3 Strengths and Weaknesses

**Figure 6:**
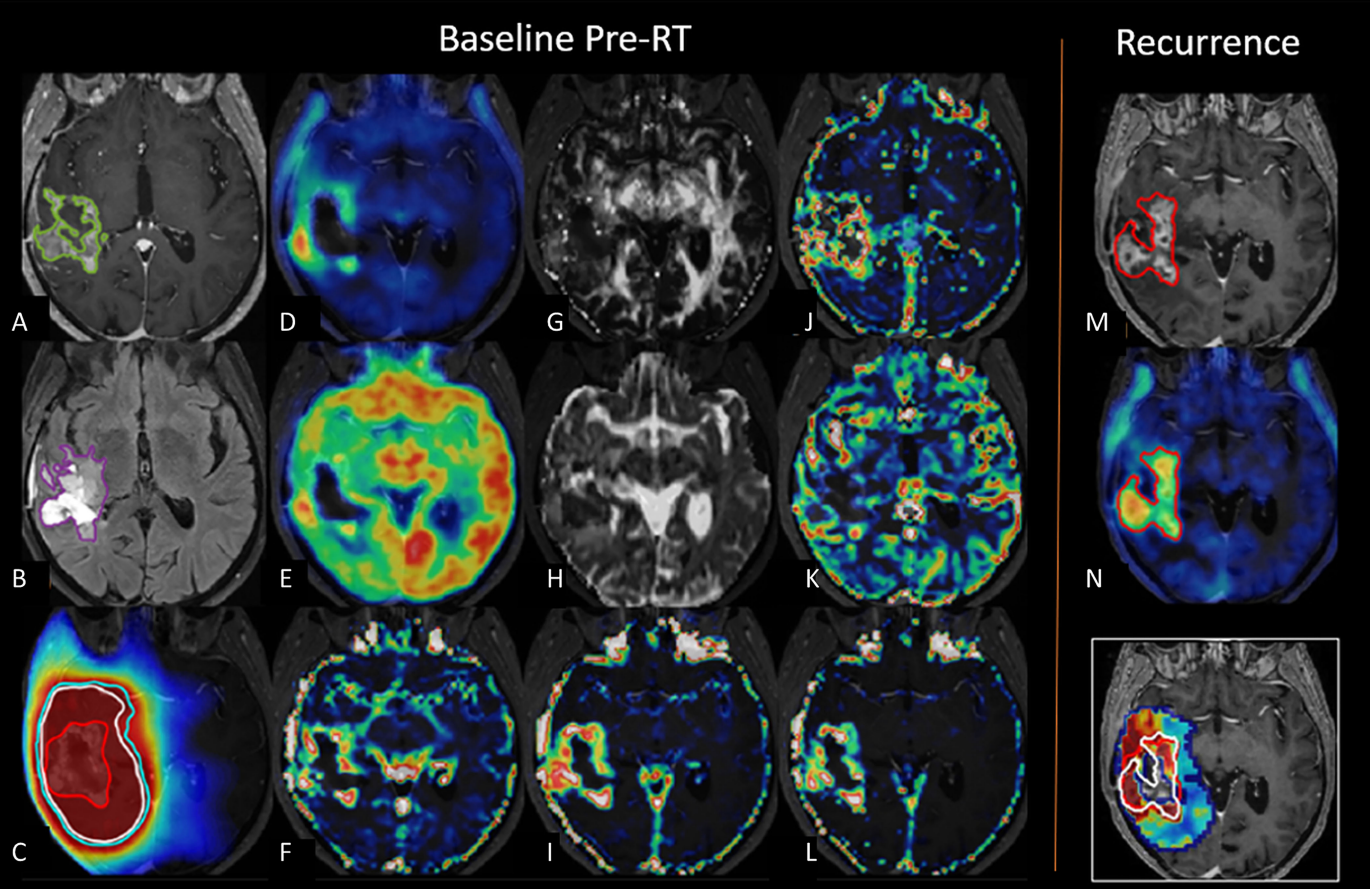
Multiparametric imaging. Example of multiparametric imaging for prediction of tumor recurrence. Baseline images prior to radiotherapy in a patient with glioblastoma show contrast-enhancing lesion (green) on **(A)** post-contrast *T*_1_-weighted images, **(B)** non-enhancing volumes (purple) on *T*_2_ fluid attenuated inversion recovery, **(C)** radiotherapy dose plan with gross tumor volume (red), clinical target volume (white), and planning target volume (cyan), **(D)** [^18^F]FET PET, **(E)** [^18^F]FDG PET, **(F)** DCE blood volume, **(G)** DTI fractional anisotropy, **(H)** DTI mean diffusivity, **(I)** DCE extravascular extra-cellular volume, **(J)** DCE mean transit time, **(K)** DCE blood flow, **(L)** DCE permeability. Follow-up imaging shows recurrent tumor in red on **(M)** post-contrast T1-weighted images and **(N)** [^18^F]FET PET imaging. Lower right image shows recurrence probability map superimposed on radiotherapy dose plan gross tumor volume (red) and actual recurrence boundary (white). Adapted with permission from (83).

The main advantage of multiparametric imaging is related to reducing both false positive and false negative results of single modalities, either by providing complementary information on biology (e.g., perfusion and metabolism) or compensating for technical limitations of one modality (e.g., limited coverage of DSC in the presence of susceptibility artifacts). Interpreting advanced multiparametric data routinely in the clinic, however, may be difficult and time consuming due to the amount and complexity of data processing and integration. **Figure 6** illustrates the complexity of multiparametric imaging. Such a challenge may be particularly true for methods requiring longitudinal data such as relative cerebral blood volume and ADC parametric response maps combinations, which appear promising in determining treatment response (84). A further limitation is, as this review has shown, a paucity of high-level evidence for individual modalities especially relating to established frameworks for technical and clinical use as well as clear thresholds with understood confidence intervals to give a robust radiological outcome; therefore, combinations of individual modalities might compound error or lead to increased uncertainty of outcome.

##### 3.2.1.4 Future Developments

One key area of development is to determine which modalities and parameters should be analyzed and integrated to give a clinically useful single diagnostic measure. One simple approach is to apply a scoring system, where each modality is rated as positive or negative, and the number of positive markers is added to a total score. One early study combining ASL, DCE, DSC, and MRS found that a score of ≥ 2 yielded a specificity of 94% as opposed to 77–84% for single modalities (85). To take into account lesion heterogeneity, one study of pseudoprgression compared to true progression applied scoring of different tumor components identified by automated voxel-based multiparametric clustering, resulting in final volume-weighted scores of the entire lesion. Applying this method in an independent test set, 87–89% of the lesions were correctly classified using the summed cluster score, compared with 76–83% using single modalities (86).

Others have applied machine learning approaches (described in more detail below) for automated voxel-wise classification of recurrence or pseudoprogression based on structural MRI, DSC, and ADC (87), or by providing maps predicting voxels where there will be downstream tumor progression (i.e., prognostic biomarkers) based on one-off multiparametric imaging prior to surgery (88) or radiotherapy (83), or through observing temporal changes in the images over time (89). A recent systematic review concluded that the integration of machine learning with multiparametric data was promising for visualization of diffusely infiltrating tumor cells before and after treatment. The review also concluded that because study cohorts are small, further studies are required to determine optimal methodology, and there is a need for larger cohorts to improve model performance (90). An advantage of machine learning is that wide data can be handled relatively easily (91) which might allow the wide spectrum of advanced imaging signatures to be captured together and thereby improve performance accuracy. However, to reiterate, a disadvantage when compared to a single modality approach is that combinations of outputs from individual modalities that are without frameworks for technical and clinical use, might compound inter-center variability and reduce generalizability considerably.

##### 3.2.1.5 PET/MRI

PET is increasingly being used in the management of brain tumors as an adjunct to MRI. **Table 2** provides an overview of the most frequently applied (or methodologically relevant) PET tracers in gliomas, grouped according to the mechanism of uptake. PET data is most frequently obtained on standalone PET/computed tomography systems and then fused to MRI, but hybrid PET/MRI systems have the advantage of allowing the simultaneous acquisition of PET and both advanced and conventional MRI within a single imaging session. Among the available tracers, only the amino acid tracers, such as [^18^F]fluoro-ethyl-tyrosine (FET), and the glucose analogue [^18^F]fluoro-deoxy-glucose (FDG) PET have been included in joint European Association of Nuclear Medicine/European Association of Neuro-Oncology (EANO) guidelines (98, 99). Amino acid tracers are generally preferred over FDG due to more specific tumor uptake (as illustrated in **Figure 6**). Repeatability of amino acid PET using [^18^F]FET has been investigated in animal models only (100). Because the main variability of PET imaging is related to the tracer and less so to the site or scanner, vendor-site-related differences are expected to be minor when consensus guidelines are followed, and PET tracers have been applied reliably in multicenter studies (101, 102).

**Table 2:** Frequently studied PET tracers used to differentiate progression from post-treatment related effects.

Target Mechanism of uptake	Tracers	Clinical evidence^a^	Sensitivity (%-%)/ Specificity (%-%)^b^	Advantages/Disadvantages
**Glucose metabolism**				
GLUT 1/3 transport and hexokinase	[^18^F]fluoro-deoxy-glucose (FDG)	+	S:43-100/40-100 M: 76-84/82-84	High availability High physiological uptake in normal structures and inflammatory foci
**Amino acid transport**				
Large amino acid transporters (LAT1 and LAT2)	[^11^C]methionine (MET)	+	S:75-91/88-100 M:93-94/82	Short half-life and need for onsite cyclotron higher uptake in inflammatory lesions.
	[^18^F]dihydroxy-phenylalanine (DOPA)	++	S: 84-100/61-100 M:86/72	Higher physiological in uptake basal ganglia
	[^18^F]fluoro-ethyl-tyrosine (FET)	++	S: 84-100/86-100 M:90-92/85-88	Added accuracy of time activity curves from dynamic imaging
				All: extensively studies and used in clinical routine, low physiological uptake
**Hypoxia**				
Trapping in hypoxic cells	[^18^F]fluoromisoinodazole (FMISO)	n.a	–	High background activity and need for delayed imaging
**Profileration**				
Thymidine kinase 1	[^18^F]fluorothymidine (FLT)	+	S:82/50	Not superior to FDG Dot not cross BBB
**Neuroinflammation**				
Mitochondrial translocator protein (TSPO)	[^11^C]PK11195	n.a.	–	[^11^C]PK11195: short half-life and need for on-site cyclotron
	[^18^F]GE-180	n.a.	–	heterogeneity and uptake in PTRE
**Perfusion**				
	[^13^]NH3	(+)	S:78-83/86	Both: freely-diffusible tracers allows quantification of perfusion
	[^15^O]H2O	n.a.	–	short half-life and need for on-site cyclotron
**Vascular endothelium**				
PSMA	[^68^Ga]PSMA			
**Cell membrane synthesis**				
Choline	[^11^C]Choline	+	S:74-92/88	[^11^C]short half-life and need for on-site cyclotron uptake in non-tumor
	[^18^F]Fluorocholine	+		Both: Uptake partially BBB dependent
**Angiogenesis**				
αvβ3 (RGD)	[^18^F]FPPRRGD2	n.a.	–	Both: Do not cross BBB
Bevacizumab	[^89^Zr]Bevacizumab	n.a.	–	
**Cancer-associated fibroblast**				
Fibroblast-activation protein	[^68^Ga]FAPI02/04	n.a.	–	Possibly BBB dependent

Selection of tracers based on recent large/systematic reviews (92–95). Footnotes: ^a^adapted from Werner et al. (95) where ++ = high diagnostic accuracy, + = limited diagnostic accuracy, (+) = limited data available, n.a. not applicable (only preliminary/no data available); ^b^Range reported in single studies (S) or meta-analyses (M) reported in (92, 93, 96, 97).

Also shown are some tracers of potential use for this indication.

**Figure 7:**
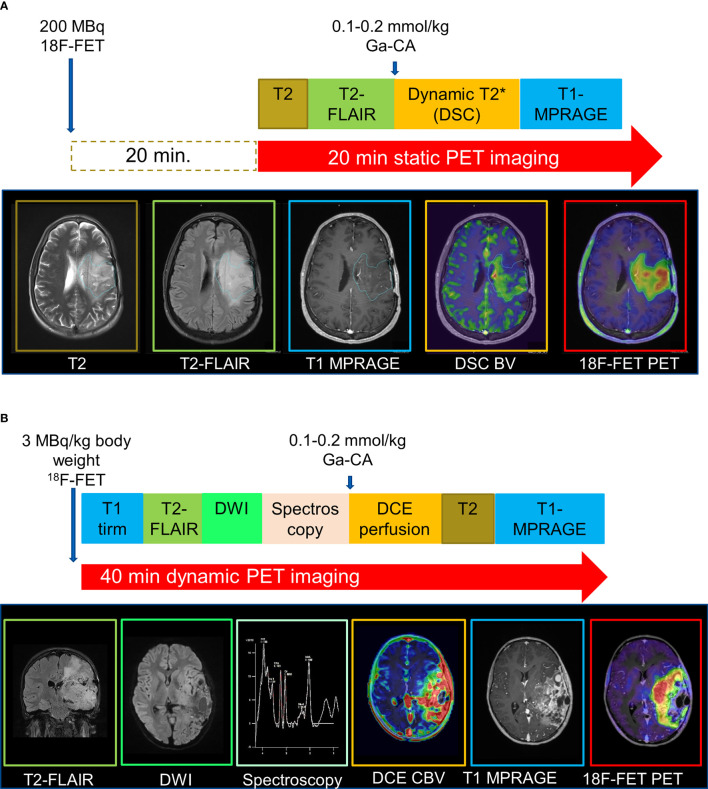
Examples of hybrid PET/MRI protocols. MRI data were acquired during acquisition of **(A)** static 20-minute or **(B)** dynamic 40-minute PET data. Adapted with permission from (103) and (104), respectively.

Several reviews have highlighted the potential of combining PET acquired simultaneously with advanced MRI by using a hybrid PET/MRI system (**Figure 7**), but the number of studies actually investigating the value of multimodal approaches in distinguishing recurrent gliomas from PTRE is limited. Recent studies combining [^18^F]FDG (105) or amino acid tracers (106–109) with DSC, DWI, and/or MRS (see **Table S2**) suggest that such multimodal imaging may provide complementary and additive information, leading to an improved overall diagnostic accuracy, but the optimal combination of modalities is not clear.

#### 3.2.2 Machine Learning and Radiomics

##### 3.2.2.1 Methodology

**Figure 8:**
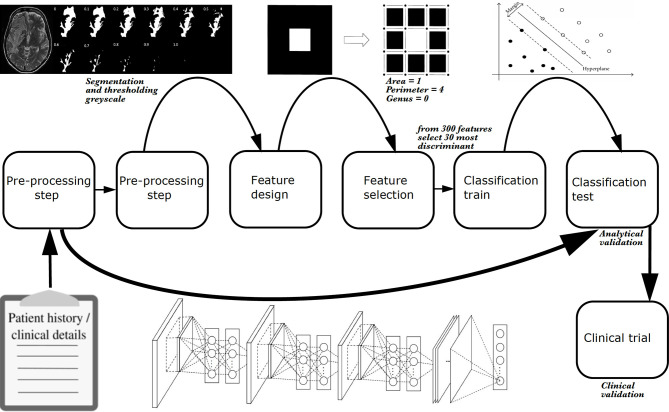
The phases of a radiomics study. Explicit feature engineering is represented by a series of boxes from left to right, starting off with pre-processing and finishing with classification of a hold-out test set. Implicit feature engineering (deep learning) is represented below these boxes by a neural network which incorporates many steps of explicit feature engineering. As with explicit feature engineering, to achieve analytical validation, classification of a hold-out test set must be performed. Once analytical validation is achieved, ideally a clinical trial tests the model to achieve clinical validation in the same way a new therapeutic agent or surgical intervention is subject to a trial. Radiomics is image based, however, additional information can be incorporated such as clinical or demographic information. All studies require some pre-processing, whether that is data cleaning or converting file format from DICOM to NIfTI, for example. With explicit feature engineering, additional pre-processing is typically required such as image segmentation. In the example shown here, hyperintense voxels associated with a grade 4 glioblastoma in a *T*_2_-weighted image are segmented as a region of interest for radiomic analysis. The mask is extracted using 11 different grey-scale thresholds to give binary combinations of black and white pixels. Thereafter, carefully designed image analysis features (or “estimated features”) can be applied to the pixels. In the example shown, these are topological descriptors of image heterogeneity (white pixel area = 1; white pixel perimeter = 4; rings subtracted from holes, i.e., genus = 0) (110). The most discriminant features can be selected using statistical or machine learning techniques, and undergo classification using a machine learning algorithm. In the example shown, a support vector machine is used (the machine learning algorithm is described as “classical” to distinguish it and other similar algorithms from deep learning algorithms), and progression (solid black dots) and pseudoprogression (empty black dots) cases are determined.

“Radiomics” (**Figure 8**) is the extraction of underlying quantitative information from the imaging dataset to develop biomarkers that may not be readily visible to individual human raters. Typically, radiomics consists of the following phases: preprocessing images, feature estimation (quantifying or characterizing the image), feature selection (dimensionality reduction to remove noise and random error in the underlying data, and, therefore, reduce overfitting), classification (decision or discriminant analysis), and evaluation (111). Evaluation in image analysis research initially consists of analytical validation, where the accuracy and reliability of the biomarker are assessed (112). Clinical validation is the subsequent clinical testing of biomarker performance, typically in a clinical trial.

Some studies have used applied statistical models, some have employed machine learning models, and many have leveraged both. The basic difference between them is that statistics draws population inferences from a sample, and machine learning finds generalizable predictive patterns (113). Recent work has made use of developments in technology to allow the use of much more complex supervised, unsupervised, and reinforcement machine learning, including the use of deep (multiple layered) neural networks, which allows automation of both feature estimation and selection steps (91).

##### 3.2.2.2 Strengths and Weaknesses

Several barriers exist in translating machine learning high-grade glioma monitoring biomarkers to the clinic (114). These predominantly relate to the requirement of large datasets that have been accurately labeled to train models. However, machine learning has some additional weaknesses. Accuracy-driven performance metrics have led to a trend towards increasingly opaque models (115), although recent developments in interpretability and explainability may help to mitigate this to some extent (116). Furthermore, linking the empirical data to a categorical analysis neglects an intrinsic ambiguity in the observed phenomena (117), which might adversely affect the intended performance (118). Also, algorithms may be unreliable due to several technical constraints: domain adaptation is currently limited, and more solutions are required to help algorithms extrapolate well to new centers. This is particularly true of advanced imaging where the lack of established frameworks for technical acquisition and clinical handling leads to spatial heterogeneity of data across hospital sites. Multi-parametric combinations of advanced imaging exacerbates the heterogeneity further and increases the challenge of model generalizability further. Robustness to unintended data, such as artifacts, is also a technical constraint that needs to be overcome. Finally, the presence of more than one pathology (e.g., abscess associated with a tumor following treatment) can also confound algorithms as these cases are scarce and often unlabeled.

Nonetheless, machine learning models have several key advantages. They require less formal statistical training given the huge developments in software (119), and the programming expertise for researchers has now been transformatively reduced, enabled by standardized implementations of open source software (120, 121). Machine learning models also have the ability to determine implicitly any complex nonlinear relationship between independent and dependent variables (119), and have the ability to determine all possible interactions between predictor variables (115).

##### 3.2.2.3 Evidence From Clinical Studies

As shown elsewhere, multiple studies have attempted to develop monitoring biomarkers to determine treatment response. Many incorporate machine learning as a central pillar of the process. A review of studies up to 2018 (91), a systematic review of studies from 2018 – 2020 (122) using PRISMA-DTA methodology and a meta-analysis from 2018–2021 (123) indicated that those taking advantage of enhanced computational processing power to build monitoring biomarker models (e.g., using deep learning methods such as convolutional neural networks) have yet to show an advantage in performance compared with machine learning techniques using explicit feature engineering and less computationally expensive classifiers (e.g., using “classical” machine learning methods support vector machine). It is also notable that studies applying machine learning to build monitoring biomarker models have yet to show an overall advantage over those using traditional statistical methods. There is good diagnostic performance of machine learning models that use MRI features to distinguish between progressive disease and diagnostic accuracy measures comprise recall = 0.61 – 1.00, specificity = 0.47 – 0.90, balanced accuracy = 0.54 – 0.83, precision = 0.58 – 0.88, F1 score = 0.59 – 0.94, and AUC = 0.65 – 0.85 (122, 123). The recent meta-analysis of ten studies showed a pooled true positive rate (sensitivity) = 0.769 (0.649 – 0.858), a false positive rate (1-specificity) = 0.352 (0.251 – 0.468) and a summary AUC-ROC = 0.765. Other pooled metrics showed derived measures of balanced accuracy = 0.706 (0.623–0.779); positive likelihood ratio = 2.220 (1.560–3.140); negative likelihood ratio = 0.366 (0.213–0.572); and diagnostic odds ratio = 6.670 (2.800–13.500) (123). It is noteworthy that the small numbers of patients included in these studies, the high-risk of bias and concerns of applicability in the study designs, and the low level of evidence given that the monitoring biomarker studies are retrospective, suggest that limited conclusions can be drawn from the data. The results show that glioblastoma treatment response monitoring biomarkers developed through machine learning are promising but are at an early phase of development and are not ready to be incorporated into clinical practice to distinguish tumor progression from PTRE. Furthermore, no practice guidelines exist for this specific application. All published studies would benefit from improvements in the methodology. Future studies would benefit from analytical validation using external hold-out tests, as well as from larger datasets to reduce overfitting.

##### 3.2.2.4 Future Developments

Advances in brain tumor database curation will facilitate integration of imaging, clinical, demographic, and molecular marker information to create large databases which will allow machine learning models to be trained and tested at a greater scale to what has occurred previously (114). The capture of large volumes of data and the inclusion of a wider spectrum of imaging phenotypes typically results in improved diagnostic performance during machine learning or statistical tasks; the relative improvement of deep learning model performance is particularly marked (124–126). For deep learning, the dependency on very large datasets can be reduced by data augmentation and transfer learning; the latter, where an already-developed model for a task is reused as the starting point for a model on a second task, is especially advantageous for medical tasks, since these pretrained models not only obviate the need for very large datasets but are less computationally expensive (116, 127, 128). One- or few-shot learning is related to this and allows classifiers to be built from very small labeled training sets (129).

Once established, incoming data from large-scale live repositories will allow ongoing refinement and assessment of outcomes. Furthermore, distributed machine learning approaches, in particular federated learning, will enable training on a large body of decentralized data (130). Federated learning is one instance of the more general approach of *bringing the code to the data, instead of the data to the code* and mitigates the fundamental problems of privacy, ownership, and locality of data. Although this technique is at the early research stage, federated learning appears to be fit-for-purpose for privacy-preserving medical applications (131, 132), and for high-grade glioma monitoring biomarkers in particular. However, the potential privacy and performance trade-off is unknown. Once established, federated learning will likely speed up the validation of the proposed methods, since fewer administrative data access requirements will be required, yet the sample will continue to be expanded by new data arriving from several sites.

### 3.3 Acceptance

#### 3.3.1 Endorsement in Guidelines

Although diagnostic accuracies of most modalities appear high enough for clinical application, and this should encourage their clinical use, acceptance in clinical guidelines is limited for a variety of reasons associated with clinical readiness, which is summarized in **Table 3**. In the recent EANO/Society for Neuro-Oncology guidelines for management of glioblastoma (144) and EANO guidelines for diffuse gliomas (145), only perfusion MRI and amino acid PET are suggested as being helpful, and they are only mentioned in the case of suspected pseudoprogression. In the 2017 modified Response Assessment in Neuro-Oncology (RANO) criteria (141), it is noted that advanced MRI techniques, such as DSC, DCE, and amino acid PET, “have shown promise but additional work is necessary to standardize these approaches and improve their sensitivity and specificity” and “issues of cost and accessibility will need to be addressed before they can be widely adopted in clinical trials.” Accordingly, the RANO criteria remain based on post-contrast *T*_1_-weighted images only (and the *T*_2_-weighted/fluid attenuated inversion recovery in 2010 RANO guidelines, albeit not quantified). In the proposed minimum imaging protocol from the Jumpstarting Brain Tumor Drug Development Coalition (143), designed to be widely applicable to a variety of MR scanners, only DWI (three b-values) is included in addition to these conventional structural sequences. DWI also has been included in the proposed minimum imaging protocol in the pediatric high-grade glioma RANO recommendations due to its widespread use and “potential benefit,” while perfusion MRI and MRS are considered experimental (149). A summary of a survey of national imaging guidelines conducted among GliMR-associated countries are included within **Table 3** (methodology and results in **Supplementary Material**). Specifically, we determined whether there are guidelines for incorporation (routine or optional) of advanced MRI techniques in clinical practice for determining treatment response in high-grade gliomas.

**Table 3:** State of development of advanced MRI techniques.

	Track & Domain^a^	Perfusion			MRS		Diffusion		CEST	PET	Criteria		
		DSC (133)	DCE (133–136)	ASL (133, 137, 138)	Single	CSI	ADC	DTI	APT (53)	AA (100, 102, 139)	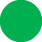	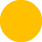	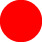
***Technical validation***													
Test-retest repeatability	T2	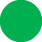	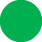	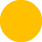	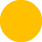	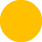	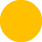	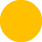	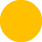	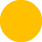	Yes, with current standard implementation	Yes, but with other implementation or patient group/animal model	None available
Cross-vendor reproducibility	T2	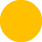	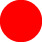	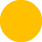	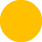	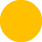	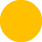	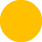	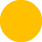	n.a.	Yes, with current standard implementation	Yes, but with other implementation or patient group	None available
Multisite reproducibility	T3	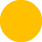	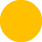	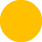	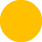	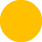	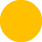	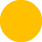	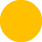	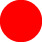	Yes, with current standard implementation	Yes, but with other implementation or patient group, phantom or analysis	None available
***Clinical evidence***													
Proof of concept in patients	C1	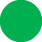	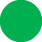	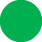	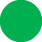	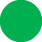	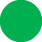	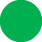	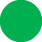	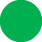	Differentiation tumor from PTRE	Differentiation tumor from normal brain	None available
Evaluated in clinical studies	C2-3	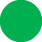	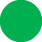	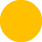	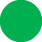	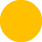	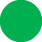	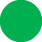	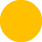	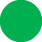	Multiple single center	Few or preliminary studies	None available
Evaluated in multi-center studies	C3	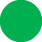	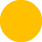	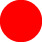	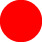	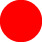	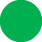	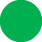	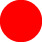	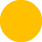	Good quality with relevant question	Small, preliminary or only method stability/not relevant question	None available
Evaluated in meta-analysis		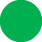	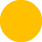	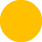	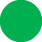	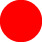	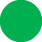	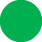	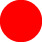	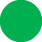	Consistent result with standard measures	Not standard measure/method, or low number studies/patients	None available
Established diagnostic accuracy, cut-offs/criteria	C3	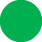	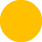	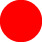	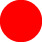	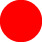	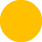	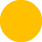	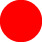	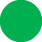	Consistent in multiple single center studies	Few or preliminary studies	None available
***Acceptance***													
Method guidelines/recommendations	T	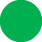	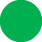	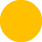	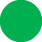	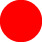	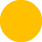	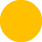	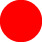	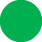	Available and updated	Available, but not updated or not specific for tumor imaging	None available
Included in clinical trial guidelines^b^		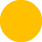	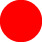	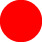	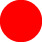	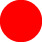	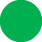	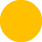	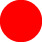	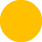	Included in suggested standard protocol	Mentioned, but clinical value uncertain	Not mentioned
Included in national imaging guideline		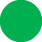	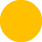	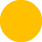	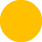	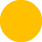	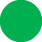	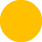	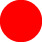	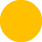	Endorsed by majority	Only endorsed but a minority	Not mentioned
Included in international clinical guidelines^c^		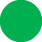	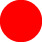	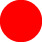	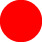	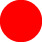	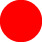	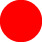	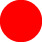	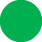	Endorsed by major international society guidelines	Mentioned, but clinical value uncertain	Not mentioned
In clinical use for brain tumor imaging^d^		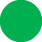	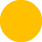	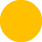	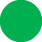	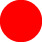	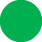	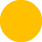	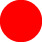	n.a.	Widely implemented (>50%)	Intermediate (<50%)	Uncommon
In clinical use for PTRE vs glioma recurrence^d^		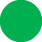	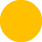	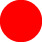	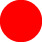	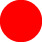	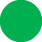	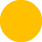	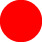	n.a.	Widely applied (>50%)	Intermediate (<50%)	Uncommon
***Implementation***													
Sequence availability	T2	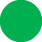	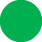	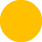	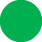	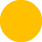	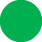	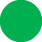	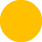	n.a.	Comparable sequence available as clinical from all major vendors	No standard implementation or only work in progress	Research sequence at singles sites
Post-processing software availability	T2	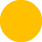	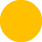	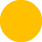	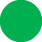	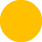	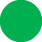	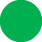	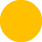	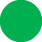	On-line scanner/reading work station with best practice implementation	Off-line, commercially available software	In-house software
Subjective ease of data acquisition (scanner operator e.g. clinical radiographer)	T2	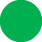	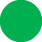	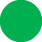	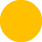	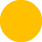	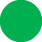	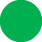	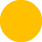	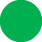	Minimal need for training	Special training/attention required	Difficult to obtain good quality data
Subjective ease of post-processing (within clinical department e.g. clinical radiologist)		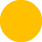	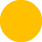	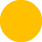	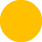	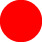	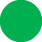	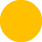	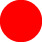	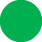	No post-processing needed	Extra processing/training needed, but not time consuming	Expert or time intensive processing required
Subjective ease of data interpretation (clinician e.g. clinical radiologist)		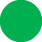	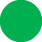	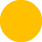	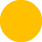	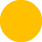	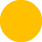	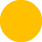	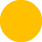	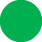	Visual reading or only simple manual steps required	Special training/expertise required	Highly specialized in single centers

^a^Imaging biomarker roadmap (140); ^b^Response assessment in neuro-oncology (RANO) (141), modified RANO criteria (142), standardized imaging protocol in clinical trials (143); ^c^Society for Neuro-Oncology (SNO) and European Society of Neuro-Oncology (EANO) consensus review on management of glioblastoma (144), EANO guidelines on diffuse gliomas (145), EANO guideline on adult astrocytic and oligodendroglial gliomas (146); ^d^European survey on advanced MRI (147), American Society of Neuroradiology survey on perfusion imaging (148).

T, technical validation; C, clinical validation; Domain 1, discovery; Domain 2, validation (lower level evidence); Domain 3, validation (higher level evidence). Also included is amino acid PET. n.a., not applicable.

#### 3.3.2 Clinical Use of Advanced MRI

Published evidence of the current use of advanced MRI in daily clinical practice is limited. European surveys have reported that advanced MRI techniques are widely available (150) and also applied to brain tumor imaging (147, 151, 152) with substantial national differences. A survey of 220 European centers (3% survey yield) showed that despite widespread availability of advanced MRI techniques, to differentiate radiation necrosis from progressive disease, perfusion imaging is used most commonly (56% of centers), whereas MRS and DWI are used rarely (6% and 5% of centers, respectively) (147). A predominantly US survey of perfusion MRI (5% survey yield) reported widespread availability for brain imaging (all indications) offered by 81% of centers, with DSC being the most frequently offered (87%) followed by DCE (41%) and ASL (35%) (148). Among those offering perfusion MRI, the most frequent indication was post-treatment evaluation of intra-axial brain tumors (87%), in particular differentiating progression from radiation necrosis (96%) or pseudoprogression (84%). The authors note that perfusion imaging is widely adopted despite the lack of reimbursement and the limited support for perfusion imaging in guidelines at the time of the survey, suggesting that both the radiologist and the referring physician find value in these techniques. However, although there appears to be a wide adoption of advanced MRI, the results of the US and European surveys may be confounded by unrepresentative samples with > 95% of non-responders. A UK survey of post-operative imaging of all neuro-oncology centers (100% survey yield) showed that most centers (> 80%) included DWI in the standard protocol, while other advanced MRI techniques (DSC, DCE, or MRS) were applied routinely by only 10% of centers during follow-up, and in selected cases where there was possible pseudoprogression by 35% (153). Of interest, neuroradiologists were the main advocates for the use of advanced imaging, while neuro-oncologists were more likely to suggest that further evidence is needed.

## 4 Conclusion

The biochemical composition of high-grade gliomas is markedly different from healthy brain tissue. MRS allows the simultaneous acquisition of an array of metabolic alterations with Cho-based ratios appearing to be consistently discriminatory in treatment response assessment, although challenges remain in this technique despite it being mature. Promising directions relate to ultra-high field strengths and high-resolution MRSI, 2HG analysis, and the use of non-proton nuclei. Labile protons on endogenous proteins can be selectively targeted with CEST to give high-resolution images. The body of evidence for clinical application of APT imaging has been building for a decade, but more evidence is required to confirm the use of CEST as a monitoring biomarker. Multiparametric methodologies, including the incorporation of nuclear medicine techniques, combine probes measuring different tumor properties. Although potentially synergistic, the limitations of each individual modality can also be compounded, particularly in the absence of standardization. Machine learning requires large datasets with high-quality annotation; currently, there is low-level evidence for monitoring biomarker clinical application.

In conclusion, advanced MRI techniques show huge promise in treatment response assessment. The clinical readiness analysis highlights that most monitoring biomarkers require standardized international consensus guidelines, with more facilitation regarding technique implementation and reporting in the clinic. The benefit of technique standardization will be multiplied in terms of multiparametric imaging and will also help leverage the enormous potential of machine learning tools.

## Author Contributions

Authors TB and OH served as overall editors. The individual sections were drafted by: Introduction (TB), DSC-MRI (KS and MÁ-T), DCE-MRI (OH), ASL (PF and VK), diffusion techniques (RN, FR, and VK), spectroscopy (ECW and GH), CEST (EAHW), multiparametric imaging (OMH), clinical readiness (OH), radiomics (TB), discussion (TB). All authors have contributed to the conception of the two parts of the article, revised them critically and approved the submitted versions.

## Funding

This publication is part of the COST Action CA18206 Glioma MR Imaging 2.0 (www.glimr.eu), supported by COST (European Cooperation in Science and Technology), www.cost.eu. GliMR provided travel and accommodation for members who had travelled to early networking meetings.

- KS: National Institute of Health/National Cancer Institute R01 CA255123, U01 CA176110, UG3 CA247606, Medical College of Wisconsin Cancer Center.
- GH: Austrian Science Fund grant KLI-646.
- ECW: The Dutch Research Council (NWO) Talent Programme Veni: 18144
- EAHW: The Dutch Research Council (NWO) Talent Programme Veni: 91619121
- PF: The Portuguese Foundation for Science and Technology (FCT) Grant UIDB/50009/2020.
- RN: Babes-Bolyai University, Grant GTC No. 35277/18.11.2020.
- TB: The Wellcome/EPSRC Centre for Medical Engineering [WT 203148/Z/16/Z].
- MÁ-T: The ALBATROSS project (National Plan for Scientific and Technical Research and Innovation 2017-2020 and DPI2016-80054-R (Programa Estatal de Promoción del Talento y su Empleabilidad en I+D+i).

## Conflict of Interest

KS: Ownership interest in IQ-AI Ltd and financial interest in Imaging Biometrics LLC. TB speaker’s bureau for AbbVie and Siemens Healthineers.

The remaining authors declare that the research was conducted in the absence of any commercial or financial relationships that could be construed as a potential conflict of interest.

## Publisher’s Note

All claims expressed in this article are solely those of the authors and do not necessarily represent those of their affiliated organizations, or those of the publisher, the editors and the reviewers. Any product that may be evaluated in this article, or claim that may be made by its manufacturer, is not guaranteed or endorsed by the publisher.
