# Supplementary material for: High-Grade Glioma Treatment Response Monitoring Biomarkers: A Position Statement on the Evidence Supporting the Use of Advanced MRI Techniques in the Clinic, and the Latest Bench-to-Bedside Developments. Part 2: Spectroscopy, Chemical Exchange Saturation, Multiparametric Imaging, and Radiomics
Source: Front Oncol. 2022 Feb 28;11:811425. doi: 10.3389/fonc.2021.811425 (PMC8948428; doi:10.3389/fonc.2021.811425)
Supplement: Supplementary file 1 [file DataSheet_1.zip › Supplementary Material - Part 2.docx]

Supplementary Material: Part 2

# **Supplementary Tables**

**Table S1.** Overview of CEST studies for post-treatment imaging

| **Authors** | **Type** | **Patient group** | **Field strength** | **CEST technique** | **CEST metric** | **Threshold for progression?** | **Main finding** |
| --- | --- | --- | --- | --- | --- | --- | --- |
| Park et al. 2016(1) | Retro | 21 GBMs scanned after CCRT or RT alone, progression determined by imaging alone and histopathology of 7 re-resected patients. | 3 T | 3D GRE EPI, B_1_ = 1 µT, single Gaussian pulse 70 ms, duty cycle 50%. 29 frequency off-sets ± 5 ppm. Whole brain coverage (8 m 40 s acquisition time) | MTR_asym,_ at 3.5 ppm, APT90: 90% histogram cut-off in solid portion of tumor after 3 pool fitting of Direct Saturation, Magnetisation Transfer and APT | Average of two readers: 1.9 % | APT-weighted CEST showed to be better than MRS in distinguishing tumor progression compared to post-treatment related effects. |
| Park et al. 2016(2) | Retro | 65 GBM patients treated with CCRT, and scanned with APT at first enhancement post-treatment on T1w scan. | 3 T | 3D GRE EPI, B_1_ = 1 µT, single Gaussian pulse 70 ms, duty cycle 50%. 29 frequency off-sets ± 5 ppm. Whole brain coverage (8 m 40 s acquisition time) | MTR_asym,_ at 3.5 ppm, APT90: 90% histogram cut-off in solid portion of tumor after 3 pool fitting of Direct Saturation, Magnetisation Transfer and APT | non-given, but treatment effect here 1.4% on average, tumor progression 4.0 on average. (Higher values than in (1)) | APT-weighted had added value to perfusion weighted imaging to distinguish treatment effect from progression. |
| Ma et al. 2016(3) | Not clear, likely retro | 32 GBMs, 3 months after CCRT. Progression was determined radiologically after 6 months. | 3 T | 3D GRE & SE acquisition, B_1_ = 2 µT, 4 pulses of 200 ms, 6 off-set acquisition, 15 slices, 10 min acquisition | MTR_asym,_, at 3.5 ppm. Calculate APT_mean_ and APT_max_ | APT_mean_ 2.42%; APT_max_ 2.54%. | Increased APT in tumor progression compared to pseudoprogression. |
| Meissner et al. 2019(4) | Pro | 12 GBM patients treated with CCRT | 7 T | 2D GRE acquisition, 140 Gaussian pulses (15ms, 60% Duty cycle), Two B_1_s (0.6 and 1.0 µT) used for B_1_ correction, nominal values for B_1_ of 0.6 used for further analysis. 59 offsets from -500 to 500 ppm. Include *T*_1_ mapping in acquisition | Calculate AREX - and then use dns-APT and NOE | none given | rNOE mediated CEST imaging at 7 T allowed for discrimination of responders and non-responders immediately after the end of CCRT. No differences found at later time points nor for APT post treatment. Pre-treatment dns-APT was higher for non-responders than for responders. |
| Yao et al. 2019(5) | Pro | 11 GBM patients treated with bevacizumab | 3 T | CEST-EPI / CEST-SAGE-EPI, 3 x 100 ms gaussian pulse with B_1_ = 6 µT, duty cycle of 50%, 1 to 5 slices. 29 off-sets densely sampled around −3.5 to −2.5 ppm, −0.3 to +0.3 ppm, and +2.5 to +3.5 ppm, with increments of 0.1 ppm. | MTR_asym_ at 3.0 ppm | None given | Decreased pH-weighted CEST signal in enhancing and non-enhancing areas, related to increased acidity due to bevacizumab. In 9 out of 11 patients the site of later recurrence has remaining increased pH-weighted CEST compared to contralateral side directly post-treatment. |
| Mehrabian et al. 2018(6) | Pro | 19 GBM patients, scanned at the start and several moments in and after CCRT. | 3 T | Single slice, fast field echo acquisition. B_1_ = 0.522 µT, 4 block-shaped pulses of 242.5 ms duration. Acquired from -5.9 to 5.9 ppm, with increments of 25 Hz steps. | Calculate MTR_asym_, and LD (constant MT + 4 lorentzian line shapes). DS is estimated based on *T*_1_/*T*_2_ mapping. | None given | Change in first two weeks. MTR amide and MTR NOE are best predictors for progression. Note: non-progressors 14 days after treatment have increased APT signal compared to baseline scans, while progressors have no change (and start from a higher baseline). Find no significant differences in progression compared to post-treatment related effects in APT-weighted / NOE. |
| Park et al. 2021(7) | Pro | 36 GBM patients treated with postoperative CCRT or RT, with a radiation dose ranging from 45 to 70 Gy; and subsequent development of a new or enlarging region of contrast enhancement within the radiation field after CCRT or RT. | 3 T | 3D GRASE acquisition, 15 slices. B_1_ = 2 µT, 4 x 200ms block-shaped pulses. 6 ppms acquired (3, 3.5, 4.0 ppm, + and -). | MTR_asym_ at 3.5 ppm. To determine average APT-weighted in one ovoid region, avoiding cysts and necrosis, in tumor area best representing APT-weighted value. | 2.1 % | APT alone showed the best prediction of tumor progression compared to all other single modalities tested. Adding APT to all other parameters significantly improved distinction. |
| Liu et al. 2019(8) | Pro | 30 glioma patients, completed RT or CCRT alone. Scanned at first enhancement after treatment. Follow-up was either histology or 6 months | 3 T | 2D, SE EPI, 3 x 400 ms saturation, B_1_ = 1.5 µT, 31 frequency off-sets, from +6 to – 6 ppm, | MTR_asym_ at 3.5 ppm | None given (Recurrence on average 2%, while treatment effects are 0%) | CBF and APT were comparable, both individually better than conventional. Combination most discriminant. |
| Park et al. 2018(9) | Retro | 31 grade III-IV gliomas after CCRT or RT, scanned within 150 days of initial surgery on average | 3 T | GRE acquisition, 15 slices, continuous saturation due to two RF coils giving alternating, 40 x 50 ms Gaussian pulses at B_1_ of 2 µT). Frequency offsets at ± 2.7, ± 3.5 and ± 4.3 ppm, and at -1560 ppm. Total scan time 7 mins. | MTR_asym_ at 3.5 ppm, and then APT90 (90% cut-off of histogram, solid portion of tumor) | 1.8 % for reader 1, 2.0% for reader 2 | APT90 showed better diagnostic performance than TNR90 in high-grade recurrence. |
| Harris et al. 2015(10) | Pro | 20 patients, scanned at 3 time points: Baseline, postsurgical and prior to CCRT. | 3 T | CEST-EPI / CEST-SAGE-EPI, 3 x 100 ms Gaussian pulse with B_1_ = 6 µT, duty cycle of 50%, 1 to 5 slices. 29 off-sets densely sampled around −3.5 to −2.5 ppm, −0.3 to +0.3 ppm, and +2.5 to +3.5 ppm, with increments of 0.1 ppm. | MTR_asym_ at 3.0 ppm | None given | Increased CEST contrast in areas where later recurrence takes place, suggestive of more acidic tumor tissue being present. More acidic lesions at baseline had shorter time to progression. |
| Paech et al. 2019(11) | Pro | 26 patients (22 wildtype, 3 IDH mutation, 1 not specified). | 7 T | 2D GRE acquisition, single slice, 150 Gaussian pulses (15 ms, 60% Duty cycle), Two B_1_s (0.6 and 0.9 µT) used for B_1_ correction, nominal values for B_1_ of 0.6 used for further analysis. 59 frequency offsets from -500 to 500 ppm. Include *T*_1_ mapping in acquisition. | Calculate AREX, and then use dns-APT and NOE | None given | dns-APT signal, pre-treatment correlates with grade and OS/PFS. NB: Note that they only measure signal pre-treatment, not during or after to differentiate between progression and post-treatment related effects. |
| Regnery et al. 2018(12) | Pro | 20 patients (12/8 male/female, 19 IDH wildtype). All were scanned pre-treatment only. | 7 T | 2D GRE acquisition, single slice, 150 gaussian pulses (15 ms, 60% Duty cycle), Two B_1_s (0.6 and 1.0 µT) used for B_1_ correction, nominal values for B_1_ of 0.6 used. 59 offsets from -500 to 500 ppm. Include *T*_1_ mapping in acquisition. | Calculate AREX, and then use dns-APT and NOE | dns-APT 2.3 ; NOE-LD (decreased for early progessors) 10.89 ; MTR_asym_ NOE-weighted (decreased for early progressors) -5.23. Note: these thresholds are for pre-treatment values. | NOE-LD and NOE-weighted MTR_asym_ and dns-APT signal, measured pre-treatment, correlate with OS/PFS. Note: only measure signal pre-treatment, not during or after to differentiate between progression and post-treatment related effects. |
|  |  |  |  |  |  |  |  |

Abbreviations: AREX = apparent exchange dependent relaxation, CCRT = radiation therapy plus concomitant temozolomide chemotherapy,  dns-APT = downfield-NOE-suppressed APT, GBM = glioblastoma, HGG = high-grade gliomas, Pro = prospective, PSP = pseudoprogression, PTRE = post-treatment-related effects, Retro = retrospective, RN = radiation necrosis, rNOE = relayed nuclear Overhauser enhancement, RT = radiotherapy. Note: WHO grade nomenclature for studies before 2021 WHO

**Table S2. Multiparametric imaging studies of recurrent gliomas***

| Paper | WHO grade | | n patients /  lesions | Progression  compared to: | | Modality | Sensitivity | Specificity | AUROC | | Accuracy as  reported in study |
| --- | --- | --- | --- | --- | --- | --- | --- | --- | --- | --- | --- |
| Brendle | 90 HGG | | 131 scans | Stable disease | | FET PET | 79 | 81 |  | | 79 |
| 2021(13) | 32 LGG | |  | /remission | | DSC | 61 | 83 |  | | 64 |
|  | 9 other | |  |  | | MRS | 52 | 57 |  | | 53 |
|  |  | |  |  | | Multiparametric | 92 | 76 |  | | 89 |
| Lohmeier | 40/42 HGG | | 42 | PTRE | | FET PET | 81 | 60 | 81 | |  |
| 2019(14) |  | |  |  | | ADC | 62 | 100 | 82 | |  |
|  |  | |  |  | | FET+ADC | 97 | 60 | 90 | |  |
| Werner | HGG | | 48 | PTRE | | FET PET | 100 | 79 | 89 | | 83 |
| 2019(15) |  | |  |  | | ADC | 60 | 71 | 73 | | 66 |
|  |  | |  |  | | FET+ADC | 67 | 94 |  | | 89 |
| Pyka | 44/47 HGG | | 47/63 | PTRE | | FET PET | 80 | 85 | 86 | |  |
| 2018(16) |  | |  |  | | DSC | 60 | 77 | 71 | |  |
|  |  | |  |  | | DWI | 62 | 77 | 70 | |  |
|  |  | |  |  | | Multiparametric | 78 | 93 | 89 | |  |
| Hojjati | GBM | | 24/28 | RN | | FDG PET | 100 | 80 | 84 | |  |
| 2018(16) |  | |  |  | | DSC | 100 | 75 | 91 | |  |
|  |  | |  |  | | FDG+DSC | 100 | 100 | 100 | |  |
| Jena | Glioma, post RT | | 35/41 | PTRE | | FDG PET | 93 | 73 | 83 | | 88 |
| 2017(17) | 32/41 lesions HGG | |  |  | | MRS: Cho/Cr | 100 | 67 | 86 | | 91 |
|  |  | |  |  | | ADC | 87 | 55 | 75 | | 78 |
|  |  | |  |  | | DSC | 83 | 64 | 68 | | 78 |
|  |  | |  |  | | FDG+ADC+MRS |  |  | 94 | |  |
|  |  | |  |  | | MRS+ADC+DSC |  |  | 91 | |  |
|  |  | |  |  | | All combined |  |  | 93 | |  |
| Sogani | Glioma, post RT | | 32 | PTRE | | FET PET | 100 | 72 | 89 | | 94 |
| 2017(18) |  | |  |  | | MRS: Cho/Cr | 100 | 86 | 89 | | 96 |
|  |  | |  |  | | ADC | 91 | 44 | 73 | | 80 |
|  |  | |  |  | | DSC | 95 | 72 | 85 | | 89 |
|  |  | |  |  | | MRS+ADC+DSC | 96 | 86 |  | | 94 |
|  |  | |  |  | | All combined | 100 | 86 |  | | 97 |
| Jena | Glioma, post RT | | 26/32 | RN | | FET PET | 100 | 71 | 89 | | 94 |
| 2016(19) |  | |  |  | | MRS: Cho/Cr | 100 | 86 | 89 | | 97 |
|  |  | |  |  | | ADC | 92 | 43 | 73 | | 81 |
|  |  | |  |  | | DSC | 96 | 71 | 85 | | 91 |
|  |  | |  |  | | ADC+MRS+DSC |  |  | 90 | |  |
|  |  | |  |  | | FET+DSC+MRS |  |  | 94 | |  |
|  |  | |  |  | | All combined |  |  | 94 | |  |
| Park | II-IV | | 36 | PTRE | | APT | 84 | 73 | 87 | | 81 |
| 2021(7) |  | |  |  | | ADC | 84 | 64 | 83 | | 78 |
|  |  | |  |  | | DTI: FA | 68 | 73 | 76 | | 69 |
|  |  | |  |  | | DSC | 60 | 91 | 78 | | 69 |
|  |  | |  |  | | ADC+FA+DSC | 84 | 91 | 87 | | 86 |
|  |  | |  |  | | ADC+FA+DSC+APT | 92 | 91 | 92 | | 92 |
| Yang | HGG | | 91 | PTRE | | DSC | 73 | 90 | 90 | | 80 |
| 2020(20) |  | |  |  | | DWI | 78 | 83 | 88 | | 80 |
|  |  | |  |  | | DSC+DWI | 98 | 85 | 95 | | 92 |
| Kim | GBM | | training 61 | PSP | | ADC | 77 | 46 | 57 | |  |
| 2019(21) |  | |  |  | | DSC | 63 | 92 | 79 | |  |
|  |  | |  |  | | Radiomic all | 91 | 77 | 90 | |  |
|  |  | | validation 34 |  | | ADC | 79 | 45 | 57 | |  |
|  |  | |  |  | | DSC | 79 | 45 | 58 | |  |
|  |  | |  |  | | Radiomic all | /71 | 90 | 85 | |  |
| Razek | HGG | | 42 | PTRE | | ASL | 94 | 92 | 95 | | 93 |
| 2018(22) |  | |  |  | | DTI: FA | 94 | 82 | 95 | | 93 |
|  |  | |  |  | | DWI: MD | 81 | 79 | 81 | | 80 |
|  |  | |  |  | | All combinations | 94 | 96 | 98 | | 95 |
| Nael | GBM | | 70 | RN | | DCE: K^trans^ | 68 | 83 | 76 | | 76 |
| 2018(23) |  | |  |  | | DSC | 80 | 92 | 92 | | 86 |
|  |  | |  |  | | ADC | 65 | 78 | 70 | | 71 |
|  |  | |  |  | | DCE + DSC | 94 | 92 | 96 | | 93 |
| Anselmi | LGG/HGG | | 50 (HGG) | progression | | MRS | 71 | 90 | 79 | |  |
| 2017(24) |  | |  |  | | DSC | 80 | 100 | 90 | |  |
|  |  | |  |  | | MRS+DSC | 91 | 90 | 79 | |  |
| Wang | GBM | | 41 | PSP | | DSC | 92 | 80 | 77 | |  |
| 2016(25) |  | |  |  | | DTI (various) | 71 | 75-90 | 72-84 | |  |
|  |  | |  |  | | DTI+DSC | 76 | 95 | 90 | |  |
| Prager | HGG | | 68 | PSP | | ADC | 74 | 70 | 78 | |  |
| 2015(26) |  | |  |  | | DSC | 87 | 83 | 86 | |  |
|  |  | |  |  | | ADC+DSC | 51 | 100 |  | |  |
| Cha | GBM | | 35 | PSP | | ADC+DSC | 82 | 100 | 88 | | 94 |
| 2014(27) |  | |  |  | |  |  | |  | |  |
| Constanzo | HGG | | 29 | RN | | DSC |  |  |  | | 86 |
| 2014(28) |  | |  |  | | MRS |  |  |  | | 79 |
|  |  | |  |  | | ADC |  |  |  | | 83 |
|  |  | |  |  | | All combined |  |  |  | | 97 |
| Seeger | HGG | | 40 | PTRE | | ASL | 54 | 84 |  | | 69 |
| 2013(29) |  | |  |  | | DSC | 81 | 77 |  | | 62 |
|  |  | |  |  | | DCE: K^trans^ | 62 | 80 |  | | 69 |
|  |  | |  |  | | MRS | 70 | 79 |  | | 62 |
|  |  | |  |  | | Score >2 | 74 | 94 |  | | 83 |
| Matsusue | LGG/HGG | | 15 | RN | | DWI |  |  |  | | 87 |
| 2010(30) | post RT | |  |  | | DSC |  |  |  | | 87 |
|  |  | |  |  | | MRS |  |  |  | | 85 |
|  |  | |  |  | | Sum score |  |  |  | | 93 |
| Liu | 25 HGG | | 30 | PTRE | | CEST: APT |  |  |  | | 87 |
| 2020(8) | 5 LGG | |  |  | | ASL |  |  |  | | 90 |
|  |  | |  |  | | MRS |  |  |  | | 70 |
|  |  | |  |  | | DWI |  |  |  | | 61 |
|  |  | |  |  | | APT+ASL |  |  |  | | 93 |
| Choi | GBM | | 62 | PSP | | ASL | 79 | 64 |  | | 73 |
| 2013(31) |  | |  |  | | DSC | 82 | 68 |  | | 76 |
|  |  | |  |  | | ASL+DSC | 94 | 82 |  | | 89 |
| Park | GBM | | training 108 | PSP | | ADC | 74 | 77 |  | | 76 |
| 2015(32) |  |  |  | |  | DSC | 78 | 84 | |  | 82 |
|  |  |  |  | |  | DCE: iAUC | 78 | 81 | |  | 80 |
|  |  |  |  | |  | Cluster score | 87 | 87 | |  | 87 |
|  |  |  | validation 54 | |  | ADC | 78 | 77 | |  | 76 |
|  |  |  |  | |  | DSC | 83 | 77 | |  | 82 |
|  |  |  |  | |  | DCE: iAUC | 83 | 84 | |  | 80 |
|  |  |  |  | |  | Cluster score | 87 | 90 | |  | 87 |

Abbreviations: RN = radiation necrosis/injury, PSP = pseudoprogression, PTRE = post-treatment related effects, MET = metastases, HGG = high-grade gliomas, LGG = low grade glioma, GBM = glioblastoma, RT = radiotherapy, AUROC = area under receiver operating curve, FET = [^18^F]fluoro-ethyl-tyrosine, FDG = [^18^F]fluoro-deoxy-glucose, PET = positron emission tomography, ADC = apparent diffusion coefficient, DWI = diffusion weighted imaging, DSC = dynamic susceptibility contrast perfusion imaging , DCE = dynamic contrast enhanced perfusion imaging , ASL = arterial spin labelling, iAUC = initial area under curve, APT = amide proton transfer-weighted imaging, MRS = magnetic resonance spectroscopy DTI = diffusion tensor imaging, FA = fractional anisotropy, MD = mean diffusivity. Note: WHO grade nomenclature for studies before 2021 WHO

**2 *Multiparametric Review Additional Search Terms.**

Search terms:

Either

(multiparametric OR multimodal)

or pairwise combinations of

(positron OR pet)

(spectroscopy OR mrs )

(diffusion OR dwi OR adc OR dti)

(perfusion OR dce OR dsc OR "blood volume")

combined with

(glioma OR glioblastoma OR "brain tumor") AND (mri OR imaging OR mr) AND (recurrent OR progressive OR radiation necrosis OR pseudoprogression OR progression OR "treatment related") AND (diagnostic OR sensitivity OR accuracy) AND (mr OR mri OR magnetic) AND (english[Filter])) NOT ((review[Filter]) NOT lymphoma NOT review

**3 National Practice Guideline Survey**

*Aim:*

To assess practice guidelines of advanced MRI techniques in post treatment follow-up imaging.

*Methods:*

Participants was asked to complete a short on-line survey regarding best practice guidelines for glioma imaging and to what extent the different imaging techniques were recommended in (pre-) and post-treatment imaging, respectively. The survey featured four main questions on post-treatment advanced MRI imaging, divided into single choice, multiple choice and free text questions (Appendix S1). We used tailored design and bimodal methodology (33) to increase response rates and obtain high-quality feedback. The questionnaire was designed with feedback from academic neuroradiologists and imaging scientists. Eligible participants were chosen amongst GLIMR COST actions members as well as those known to be clinical experts in advanced MRI for high-grade glioma treatment response. An online survey tool, Google Forms (https://docs.google.com/forms), was used for data collection from July - September 2021. Each participant was invited to complete the online survey via email. Multiple individualized follow-up emails were sent to ensure completion of the survey.

*Results:*

On an intention-to-survey basis, a total of 35 out of 40 invited representatives completed the questionnaire definitively. National or regional best practice guidelines were available in 11 countries (10 national and 1 regional). Results are shown in Table S3 and Figure S1. In the remaining 24 countries no (n = 14) or only institutional (n = 10) guidelines were available. Definitive detailed information was not received from representatives, or subsequent additionally targeted representatives, from Latvia, North Macedonia, Finland, Hungary, and Iceland. However, from the information received by COST Action members there was no evidence to suggest there were national or regional guidelines in these countries.

**National Practice Guideline Survey Table S3**. Recommendations to implement advanced MRI in those countries with national or regional best practice guidelines.

|  | UK | Norway | Spain | Germany | Denmark | France | Ireland | Switzer  land | NL | USA | Sweden |
| --- | --- | --- | --- | --- | --- | --- | --- | --- | --- | --- | --- |
| Guideline type | N | R | N | N | N | N | N | N | N | N | N |
| Perfusion any | 1 | 1 | 0 | 1 | 1 | 2 | 1 | 2 | 1 | 1 | 2 |
| DSC | 0 | 1 | 0 | 0 | 1 | 2 | 1 | 2 | 1 | 1 | 2 |
| DCE | 0 | 0 | 0 | 0 | 1 | 0 | 1 | 0 | 0 | 0 | 0 |
| ASL | 0 | 0 | 0 | 0 | 0 | 0 | 1 | 0 | 0 | 0 | 0 |
| MRS any | 1 | 1 | 0 | 1 | 0 | 0 | 0 | 1 | 1 | 0 | 0 |
| SVS | 0 | 0 | 0 | 0 | 0 | 0 | 0 | 1 | 1 | 0 | 0 |
| MRSI | 0 | 1 | 0 | 0 | 0 | 0 | 0 | 1 | 1 | 0 | 0 |
| CEST | 0 | 0 | 0 | 0 | 0 | 0 | 0 | 0 | 0 | 0 | 0 |
| Diffusion any | 1 | 2 | 0 | 0 | 2 | 2 | 2 | 2 | 1 | 0 | 2 |
| ADC | 1 | 2 | 0 | 0 | 2 | 2 | 2 | 2 | 1 | 0 | 2 |
| Advanced DWI/kurtosis | 0 | 1 | 0 | 0 | 0 | 0 | 0 | 1 | 0 | 0 | 0 |
| DTI | 1 | 1 | 0 | 0 | 0 | 2 | 0 | 1 | 0 | 0 | 0 |
| PET any | 0 | 0 | 0 | 1 | 1 | 1 | 0 | 1 | 1 | 0 | 1 |
| AA PET | 0 | 0 | 0 | 1 | 1 | 0 | 0 | 1 | 1 | 0 | 1 |
| FDG PET | 0 | 0 | 0 | 0 | 0 | 0 | 0 | 1 | 0 | 0 | 0 |

Guideline type: N = national, R = regional. Recommendation: 0 = no, 1 = optional, 2 = routine. Abbreviations: DSC = dynamic susceptibility contrast perfusion imaging, DCE = dynamic contrast enhanced perfusion imaging, ASL = arterial spin labeling, MRS = magnetic resonance spectroscopy, SVS = single voxel spectroscopy, MRSI = magnetic resonance spectroscopic imaging, CEST = chemical exchange saturation transfer , ADC = apparent diffusion coefficient, DWI = diffusion weighted imaging, DTI = diffusion tensor imaging, PET = positron emission tomography, AA = amino acid , FDG = fluoro-deoxy-glucose.

**
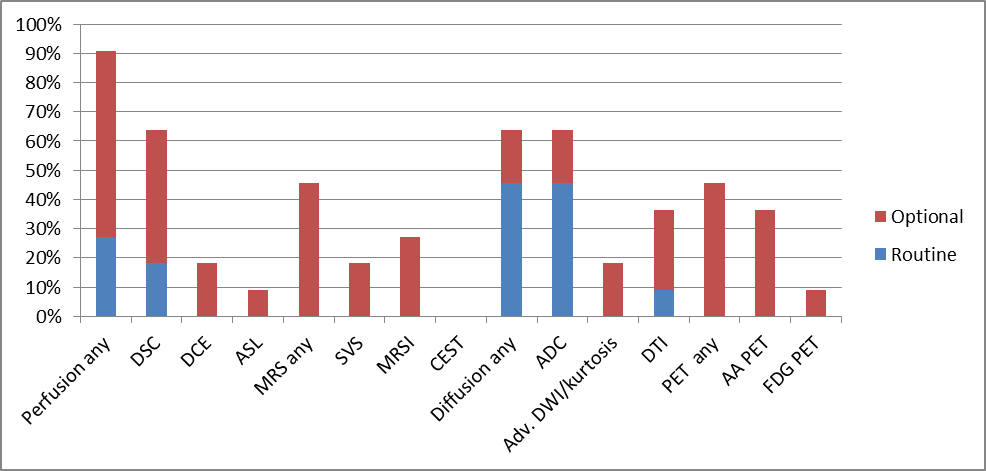
**

**Figure S1.** Bar chart showing the recommendations to implement advanced MRI in those countries with national or regional best practice guidelines.

Abbreviations: DSC dynamic susceptibility contrast perfusion imaging, DCE dynamic contrast enhanced perfusion imaging, ASL arterial spin labeling, MRS magnetic resonance spectroscopy, SVS single voxel spectroscopy, MRSI magnetic resonance spectroscopic imaging, CEST chemical exchange saturation transfer, ADC apparent diffusion coefficient, DWI diffusion weighted imaging, DTI diffusion tensor imaging, PET positron emission tomography, AA amino acid , FDG fluoro-deoxy-glucose.

**Appendix S1**

**Survey** (Section 3 background questions on pre-treatment imaging not shown here)

*What do we want you to do?*

1. Identify the national imaging guidelines relevant for imaging of high-grade gliomas. It is very important for the validity of the survey that we refer to the most relevant and updated guidelines from each country. If there are no national guideline, please use the “highest level” regional or societal guidelines. If you not sure where to find the most appropriate guidelines, we suggest to get help from a senior clinical radiologist or oncologist to assist you.
2. Go the online questionnaire found here. Fill in the questionnaire. The questionnaire is divided into sections relating first to the guidelines used, and then the use of standard and advanced imaging techniques in pre- and posttreatment imaging of high-grade gliomas.

**IMPORTANT:** This survey is not about common practice in your country/institution, but only about what is included in written guidelines.

We are aware that guidelines may be structured differently and may not specifically describe the use of each method in each situation. In that case you can state or translate into English what is recommended using the corresponding comment boxes, and we will try to fit into a common format.


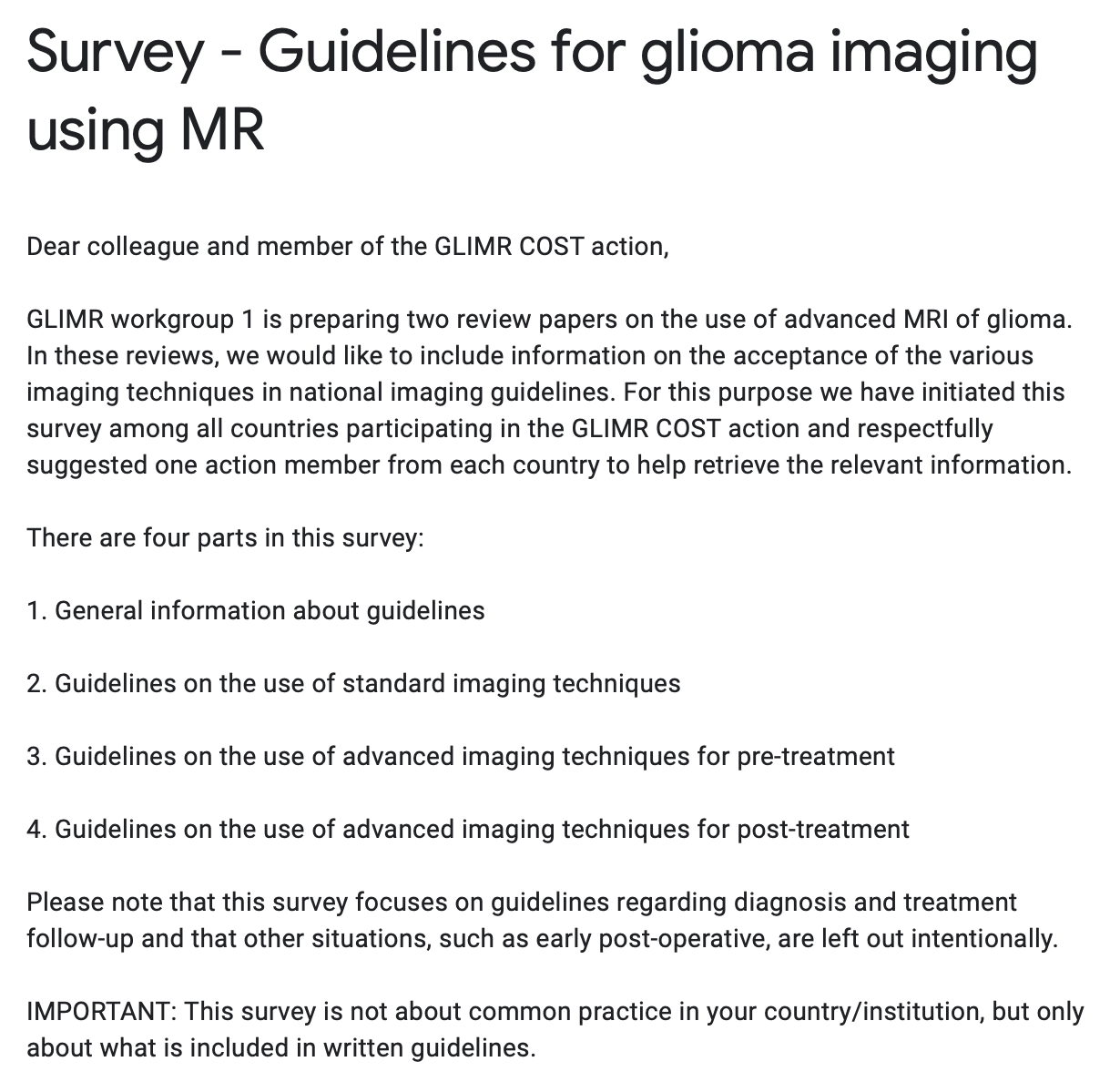


**
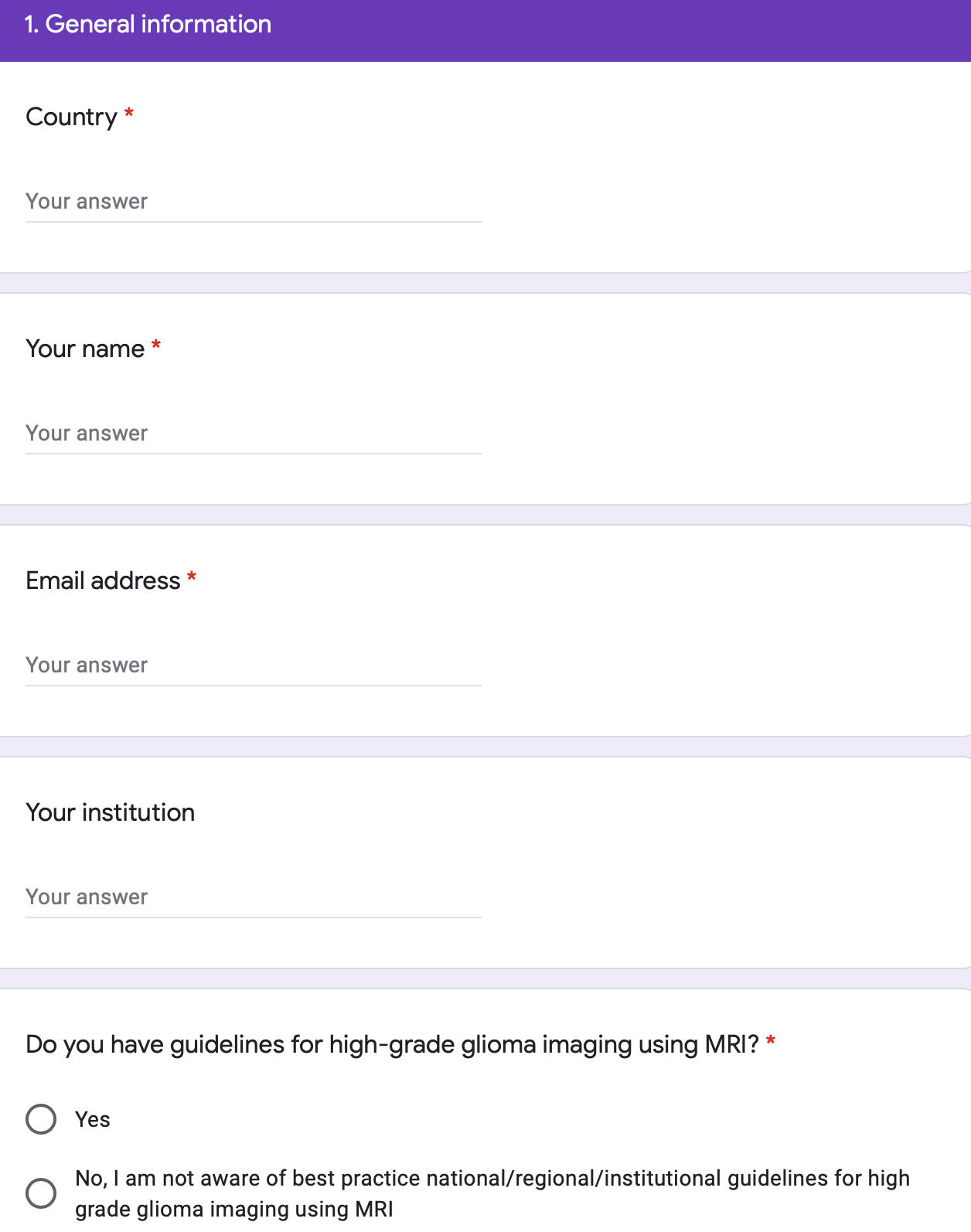
**

**\\**

**
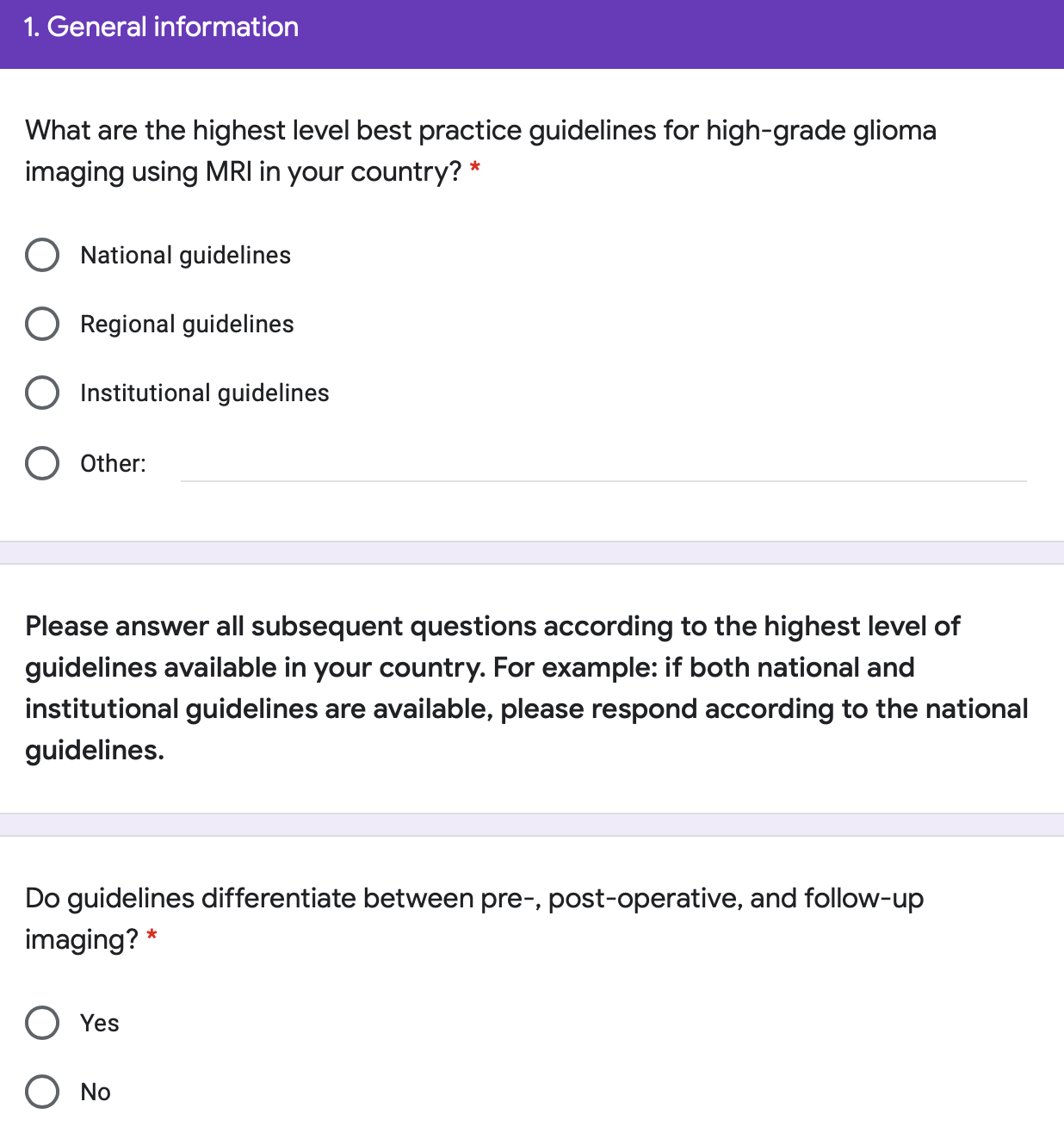
**

*
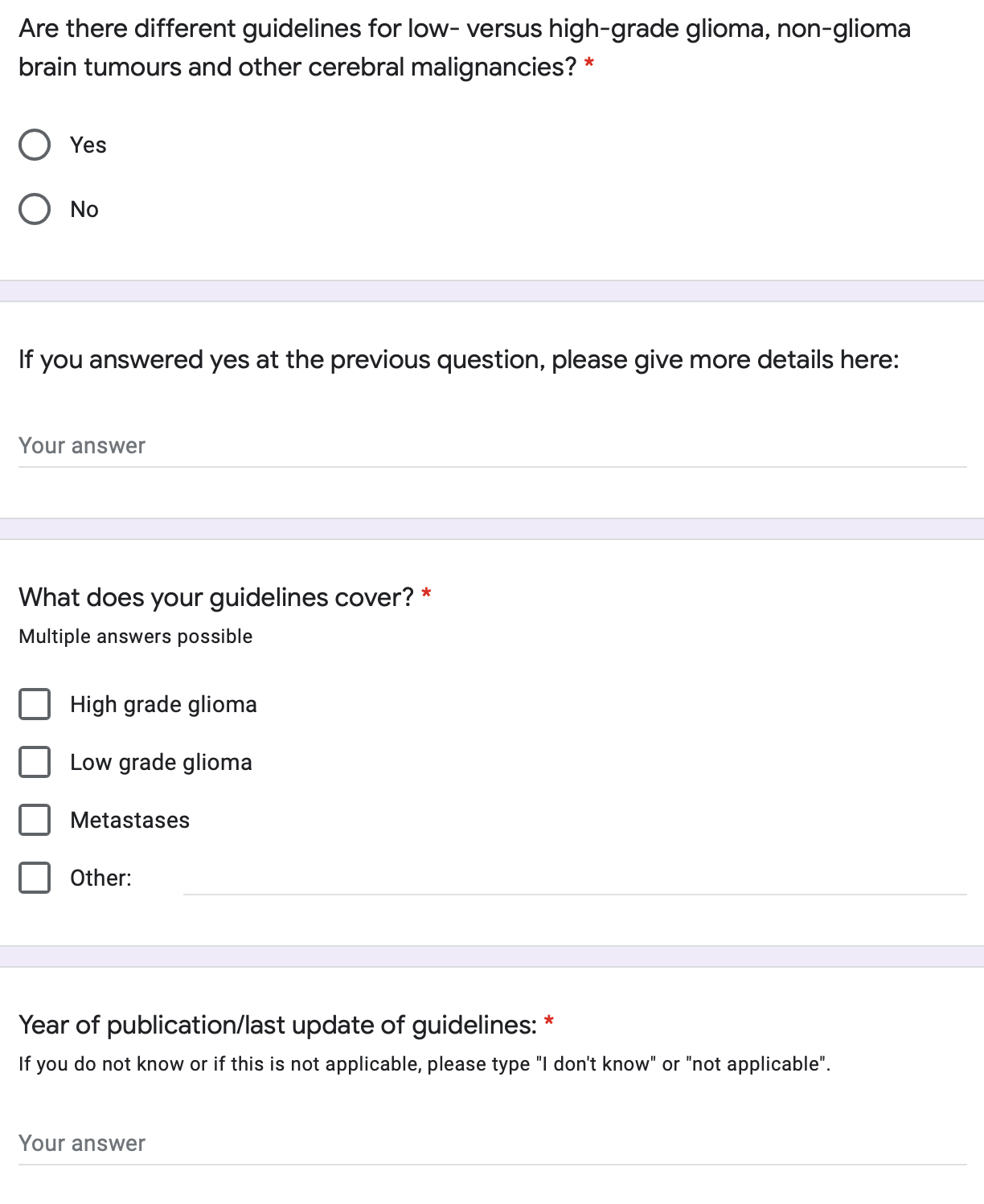
*

*
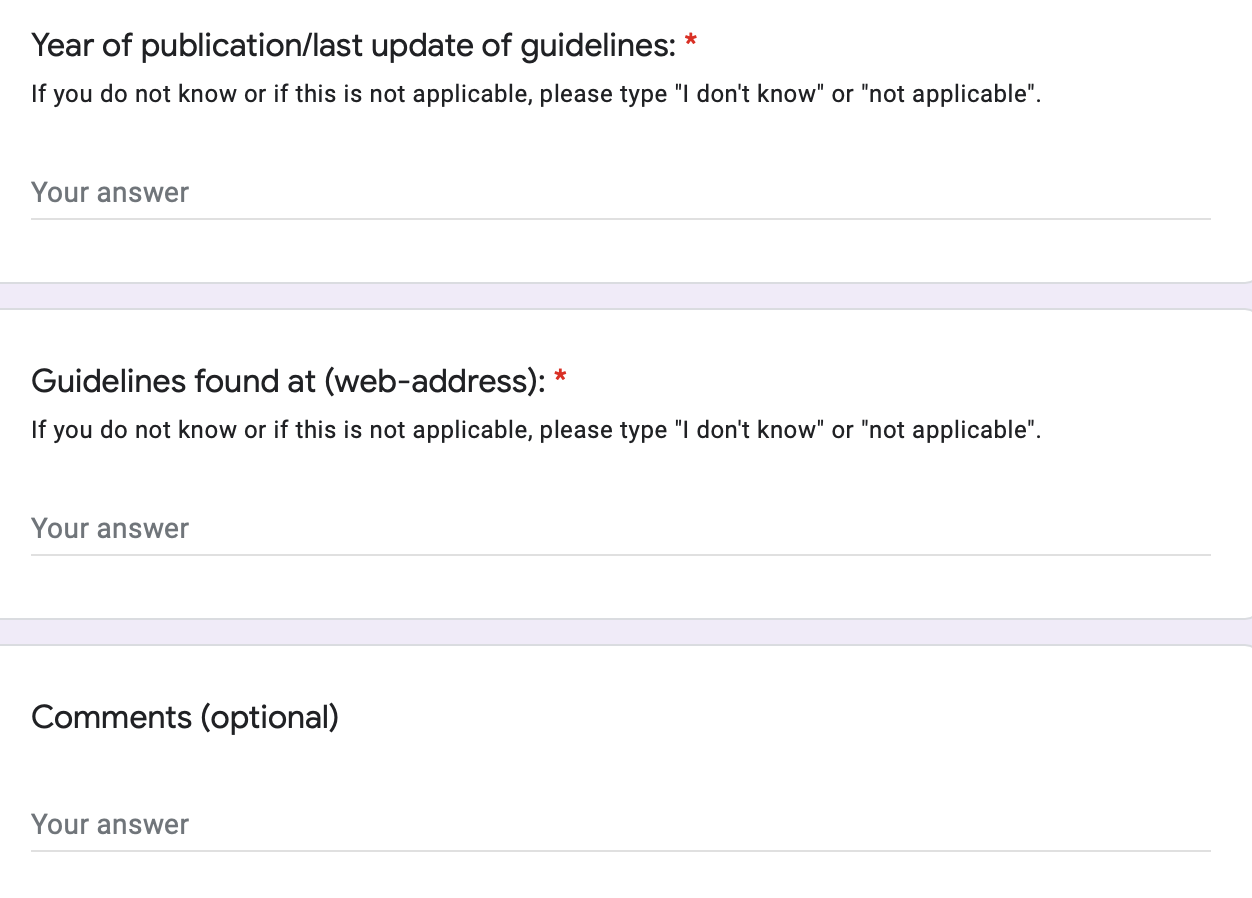
*

*
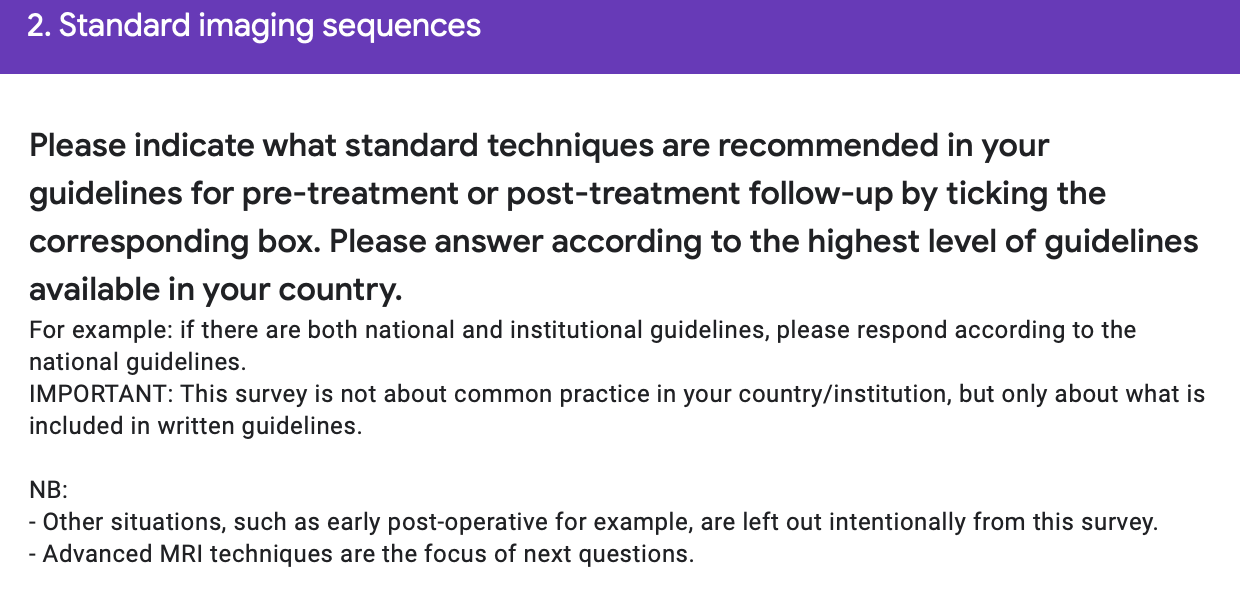
*

*
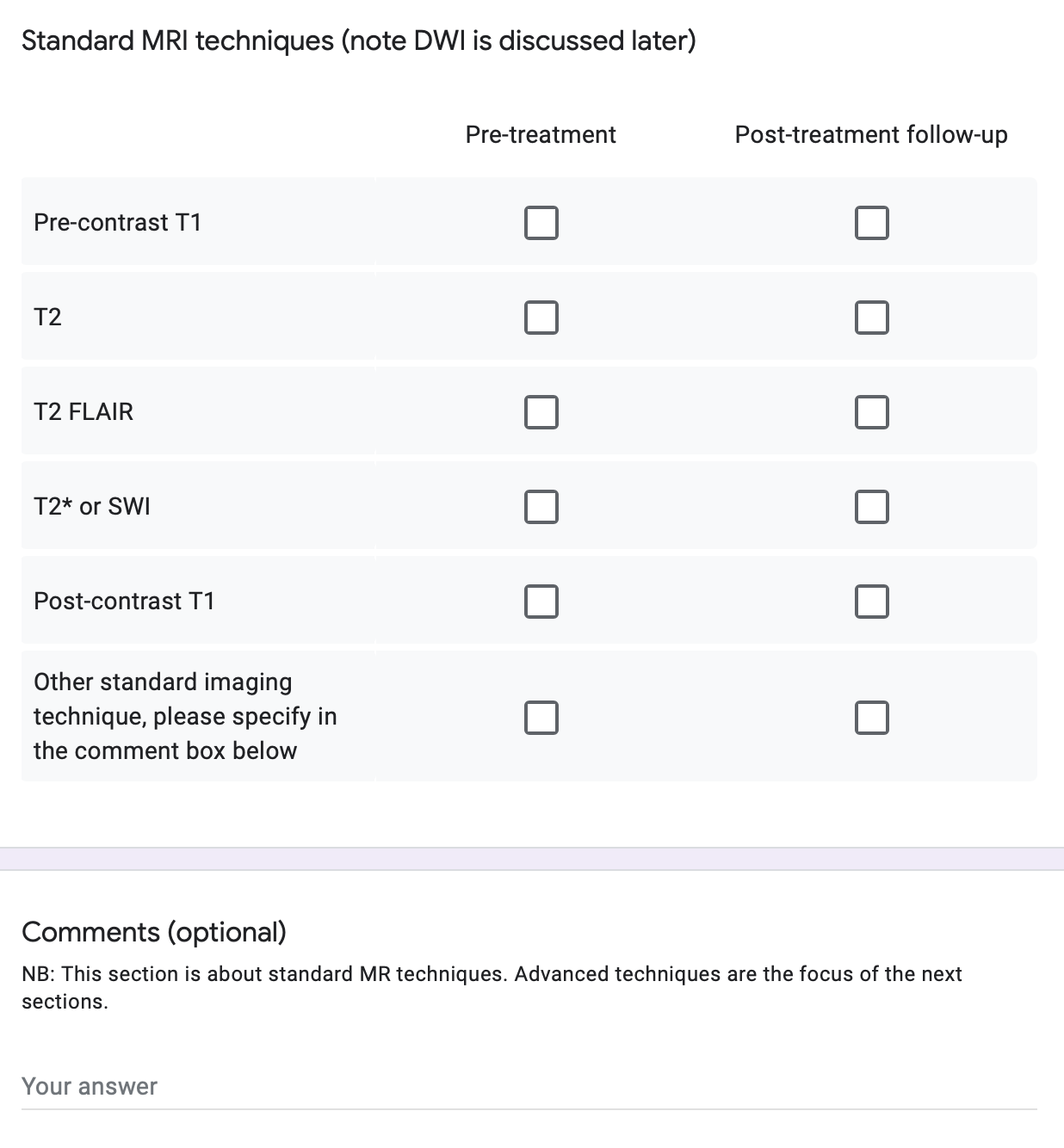
*

*
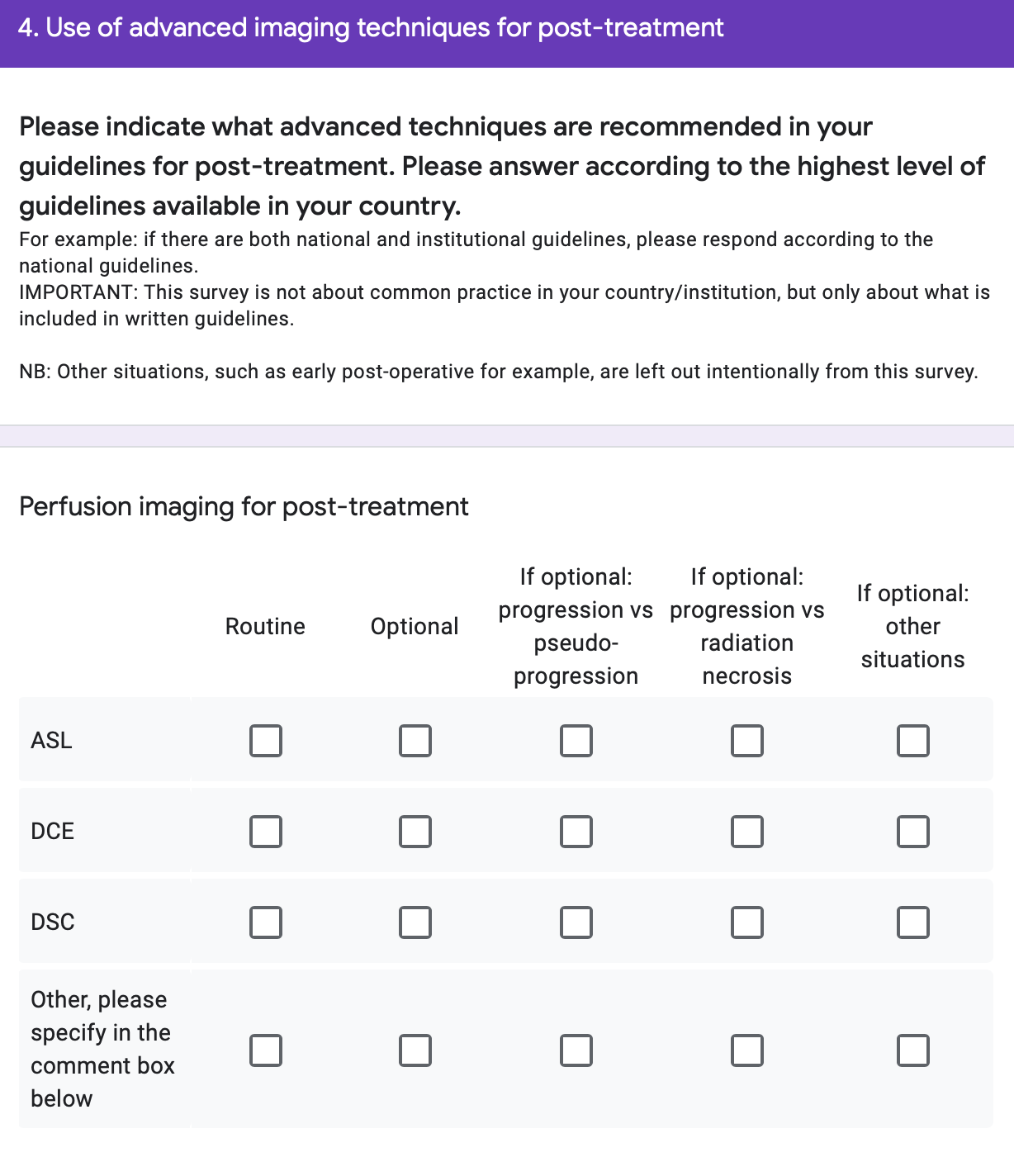
*

*
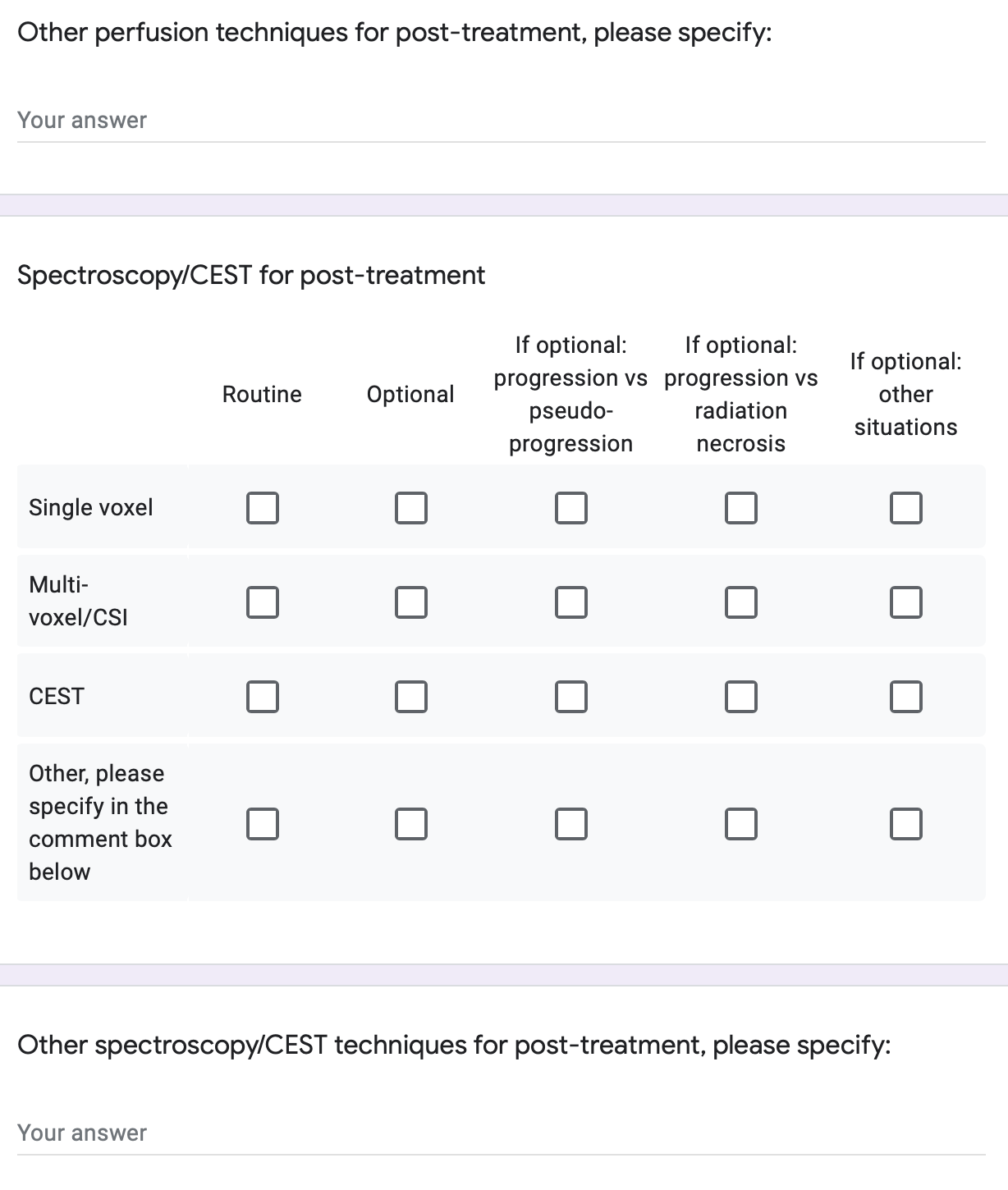
*

*
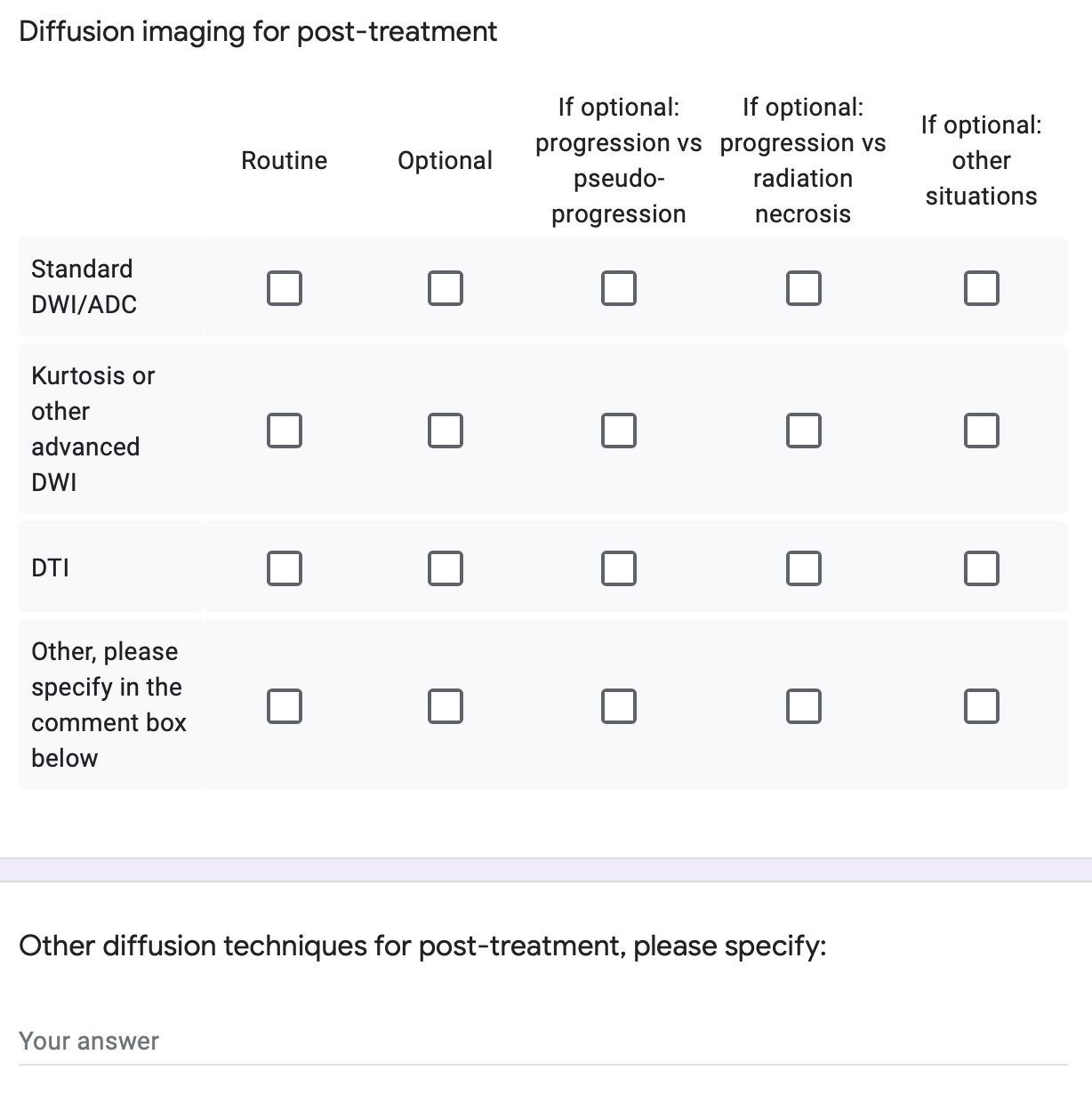
*

*
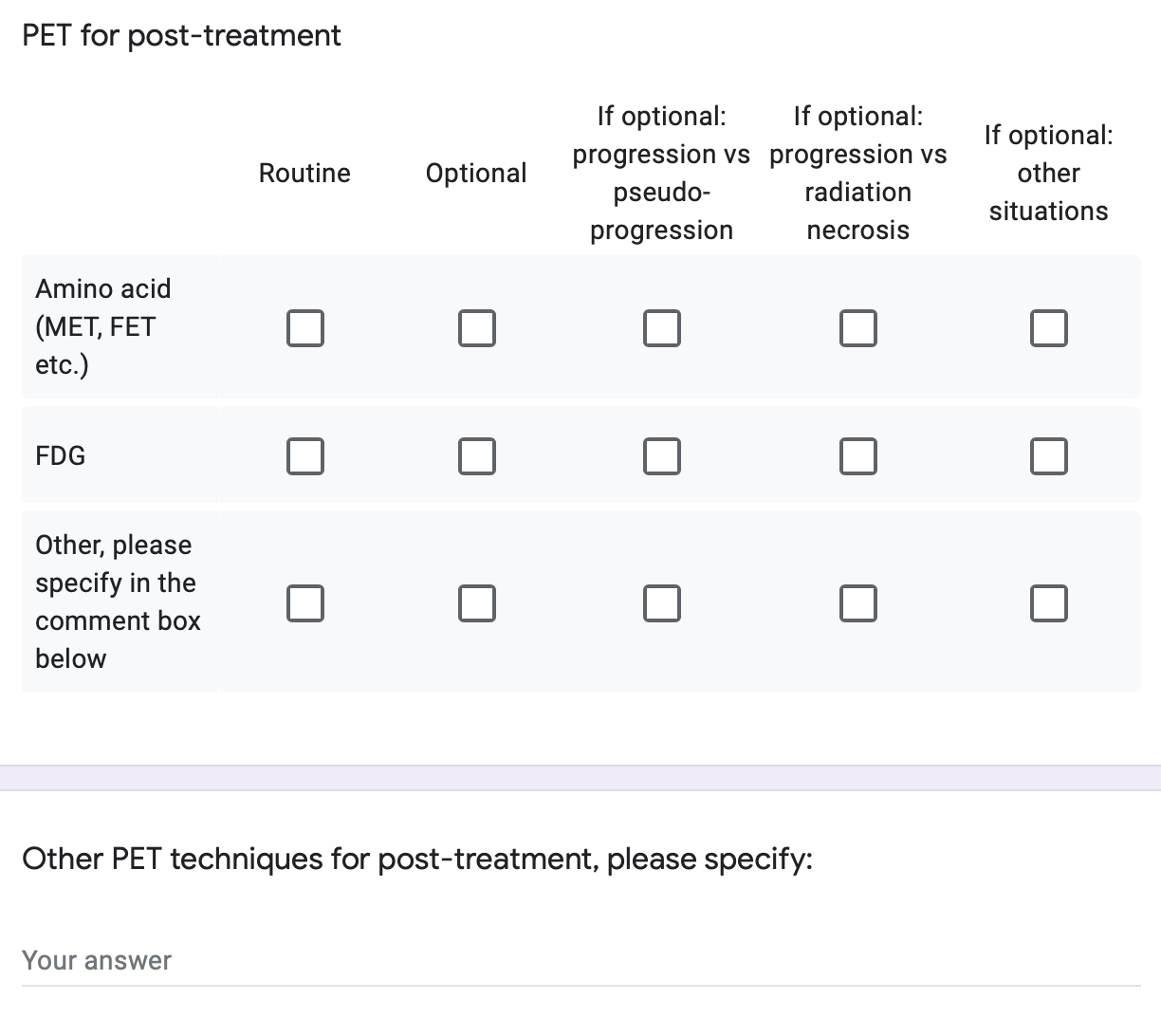
*

*
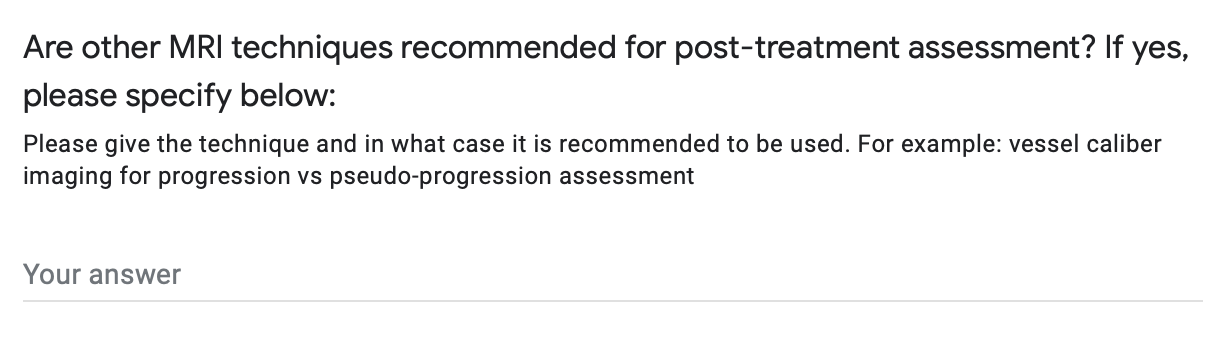
*

*
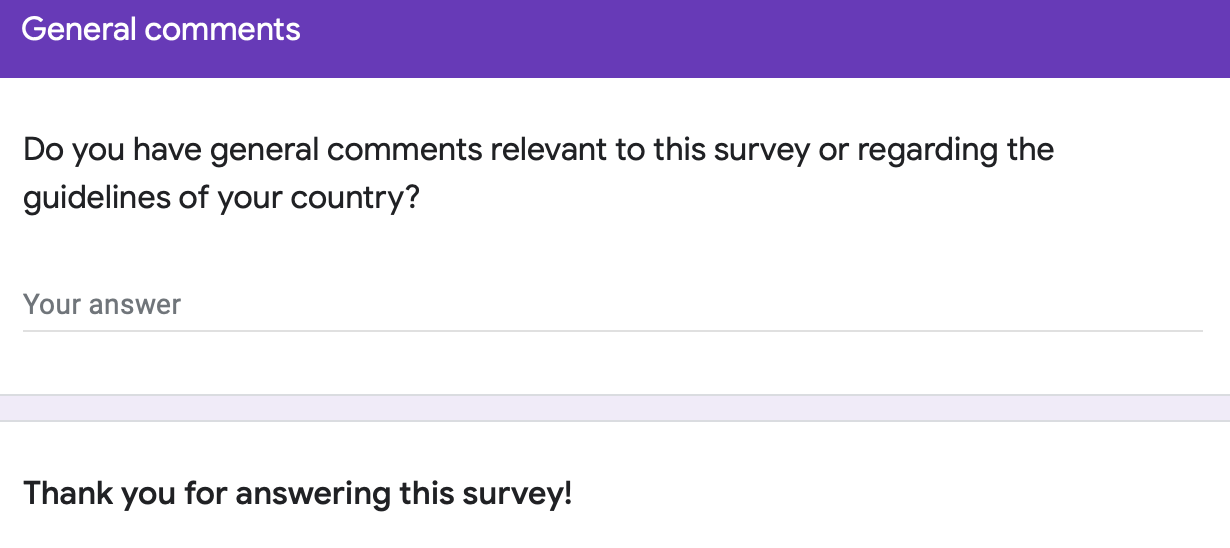
*

*Contributions:*

Survey group:

L. Hirschler (NL), T Booth (UK) , K Emblem (Norway), O.M. Henriksen (Denmark)

National expert representative:

T. Booth (UK), V.K. Katsaros (Greece), R. Grech (Malta), K. Emblem (Norway), Y. Özsunar (Turkey), M. del Mar Álvarez-Torres (Spain), M. Dakovic (Serbia), N. Sollmann (Germany), A. Šerifović-Trbalić (Bosnia and Herzegovina), E. Paal (Estonia), R. Achten (Belgium), M. Bulik (Czech Republic), V.A. Larsen (Denmark), C. Maumet (France), S. Unnikrishnan (Ireland), A. Sabisz (Poland), G. Karami (Italy), O. Islam (Canada), S. Pjević (Slovenia), J. Fierstra (Switzerland), M. Stoeva (Bulgaria), V. Keil (Netherlands), R.E. Nechifor (Romania), J. Furtner-Srajer (Austria), T. Nunes (Portugal), L.S. Hu (USA), L. Siakalli (Cyprus), M. Chmelik (Slovakia), A. Montvila (Lithuania), B. Moon (New Zealand), C. Barras (Australia), J. Wikström (Sweden) F. Borovečki (Croatia), G. Decker (Luxembourg).

The survey group would like to thank all who helped complete the questionnaire on behalf of their respective countries.

**References**

1. Park JE, Kim HS, Park KJ, Kim SJ, Kim JH, Smith SA. Pre- and Posttreatment Glioma: Comparison of Amide Proton Transfer Imaging with MR Spectroscopy for Biomarkers of Tumor Proliferation. *Radiology* (2016) **278**: doi:10.1148/radiol.2015142979

2. Park KJ, Kim HS, Park JE, Shim WH, Kim SJ, Smith SA. Added value of amide proton transfer imaging to conventional and perfusion MR imaging for evaluating the treatment response of newly diagnosed glioblastoma. *Eur Radiol* (2016) **26**: doi:10.1007/s00330-016-4261-2

3. Ma B, Blakeley JO, Hong X, Zhang H, Jiang S, Blair L, Zhang Y, Heo H-Y, Zhang M, van Zijl PCM, et al. Applying amide proton transfer-weighted MRI to distinguish pseudoprogression from true progression in malignant gliomas. *J Magn Reson Imaging* (2016) **44**: doi:10.1002/jmri.25159

4. Meissner J, Korzowski A, Regnery S, Goerke S, Breitling J, Floca RO, Debus J, Schlemmer H, Ladd ME, Bachert P, et al. Early response assessment of glioma patients to definitive chemoradiotherapy using chemical exchange saturation transfer imaging at 7 T. *J Magn Reson Imaging* (2019) **50**: doi:10.1002/jmri.26702

5. Yao J, Tan CHP, Schlossman J, Chakhoyan A, Raymond C, Pope WB, Salamon N, Lai A, Ji M, Nghiemphu PL, et al. pH-weighted amine chemical exchange saturation transfer echoplanar imaging (CEST-EPI) as a potential early biomarker for bevacizumab failure in recurrent glioblastoma. *J Neurooncol* (2019) **142**: doi:10.1007/s11060-019-03132-z

6. Mehrabian H, Myrehaug S, Soliman H, Sahgal A, Stanisz GJ. Evaluation of Glioblastoma Response to Therapy With Chemical Exchange Saturation Transfer. *Int J Radiat Oncol* (2018) **101**: doi:10.1016/j.ijrobp.2018.03.057

7. Park YW, Ahn SS, Kim EH, Kang S-G, Chang JH, Kim SH, Zhou J, Lee S-K. Differentiation of recurrent diffuse glioma from treatment-induced change using amide proton transfer imaging: incremental value to diffusion and perfusion parameters. *Neuroradiology* (2021) **63**: doi:10.1007/s00234-020-02542-5

8. Liu J, Li C, Chen Y, Lv X, Lv Y, Zhou J, Xi S, Dou W, Qian L, Zheng H, et al. Diagnostic performance of multiparametric MRI in the evaluation of treatment response in glioma patients at 3T. *J Magn Reson Imaging* (2020) **51**: doi:10.1002/jmri.26900

9. Park JE, Lee JY, Kim HS, Oh J-Y, Jung SC, Kim SJ, Keupp J, Oh M, Kim JS. Amide proton transfer imaging seems to provide higher diagnostic performance in post-treatment high-grade gliomas than methionine positron emission tomography. *Eur Radiol* (2018) **28**: doi:10.1007/s00330-018-5341-2

10. Harris RJ, Cloughesy TF, Liau LM, Prins RM, Antonios JP, Li D, Yong WH, Pope WB, Lai A, Nghiemphu PL, et al. pH-weighted molecular imaging of gliomas using amine chemical exchange saturation transfer MRI. *Neuro Oncol* (2015) **17**: doi:10.1093/neuonc/nov106

11. Paech D, Dreher C, Regnery S, Meissner J-E, Goerke S, Windschuh J, Oberhollenzer J, Schultheiss M, Deike-Hofmann K, Bickelhaupt S, et al. Relaxation-compensated amide proton transfer (APT) MRI signal intensity is associated with survival and progression in high-grade glioma patients. *Eur Radiol* (2019) **29**: doi:10.1007/s00330-019-06066-2

12. Regnery S, Adeberg S, Dreher C, Oberhollenzer J, Meissner J-E, Goerke S, Windschuh J, Deike-Hofmann K, Bickelhaupt S, Zaiss M, et al. Chemical exchange saturation transfer MRI serves as predictor of early progression in glioblastoma patients. *Oncotarget* (2018) **9**: doi:10.18632/oncotarget.25594

13. Brendle C, Maier C, Bender B, Schittenhelm J, Paulsen F, Renovanz M, Roder C, Castaneda-Vega S, Tabatabai G, Ernemann U, et al. Impact of ^18^ F-FET PET/MR on clinical management of brain tumor patients. *J Nucl Med* (2021) doi:10.2967/jnumed.121.262051

14. Lohmeier J, Bohner G, Siebert E, Brenner W, Hamm B, Makowski MR. Quantitative biparametric analysis of hybrid 18F-FET PET/MR-neuroimaging for differentiation between treatment response and recurrent glioma. *Sci Rep* (2019) **9**: doi:10.1038/s41598-019-50182-4

15. Werner J-M, Stoffels G, Lichtenstein T, Borggrefe J, Lohmann P, Ceccon G, Shah NJ, Fink GR, Langen K-J, Kabbasch C, et al. Differentiation of treatment-related changes from tumour progression: a direct comparison between dynamic FET PET and ADC values obtained from DWI MRI. *Eur J Nucl Med Mol Imaging* (2019) **46**: doi:10.1007/s00259-019-04384-7

16. Pyka T, Hiob D, Preibisch C, Gempt J, Wiestler B, Schlegel J, Straube C, Zimmer C. Diagnosis of glioma recurrence using multiparametric dynamic 18F-fluoroethyl-tyrosine PET-MRI. *Eur J Radiol* (2018) **103**: doi:10.1016/j.ejrad.2018.04.003

17. Jena A, Taneja S, Jha A, Damesha NK, Negi P, Jadhav GK, Verma SM, Sogani SK. Multiparametric Evaluation in Differentiating Glioma Recurrence from Treatment-Induced Necrosis Using Simultaneous ^18^ F-FDG-PET/MRI: A Single-Institution Retrospective Study. *Am J Neuroradiol* (2017) **38**: doi:10.3174/ajnr.A5124

18. Sogani S, Jena A, Taneja S, Gambhir A, Mishra A, D’Souza M, Verma S, Hazari P, Negi P, Jadhav GR. Potential for differentiation of glioma recurrence from radionecrosis using integrated ^18^ F-fluoroethyl-L-tyrosine (FET) positron emission tomography/magnetic resonance imaging: A prospective evaluation. *Neurol India* (2017) **65**: doi:10.4103/neuroindia.NI_101_16

19. Jena A, Taneja S, Gambhir A, Mishra AK, D’souza MM, Verma SM, Hazari PP, Negi P, Jhadav GKR, Sogani SK. Glioma Recurrence Versus Radiation Necrosis. *Clin Nucl Med* (2016) **41**: doi:10.1097/RLU.0000000000001152

20. Yang Y, Yang Y, Wu X, Pan Y, Zhou D, Zhang H, Chen Y, Zhao J, Mo Z, Huang B. Adding DSC PWI and DWI to BT-RADS can help identify postoperative recurrence in patients with high-grade gliomas. *J Neurooncol* (2020) **146**: doi:10.1007/s11060-019-03387-6

21. Kim JY, Park JE, Jo Y, Shim WH, Nam SJ, Kim JH, Yoo R-E, Choi SH, Kim HS. Incorporating diffusion- and perfusion-weighted MRI into a radiomics model improves diagnostic performance for pseudoprogression in glioblastoma patients. *Neuro Oncol* (2019) **21**: doi:10.1093/neuonc/noy133

22. Razek AAKA, El-Serougy L, Abdelsalam M, Gaballa G, Talaat M. Differentiation of residual/recurrent gliomas from postradiation necrosis with arterial spin labeling and diffusion tensor magnetic resonance imaging-derived metrics. *Neuroradiology* (2018) **60**: doi:10.1007/s00234-017-1955-3

23. Nael K, Bauer AH, Hormigo A, Lemole M, Germano IM, Puig J, Stea B. Multiparametric MRI for Differentiation of Radiation Necrosis From Recurrent Tumor in Patients With Treated Glioblastoma. *Am J Roentgenol* (2018) **210**: doi:10.2214/AJR.17.18003

24. Anselmi M, Catalucci A, Felli V, Vellucci V, Di Sibio A, Gravina GL, Di Staso M, Di Cesare E, Masciocchi C. Diagnostic accuracy of proton magnetic resonance spectroscopy and perfusion-weighted imaging in brain gliomas follow-up: a single institutional experience. *Neuroradiol J* (2017) **30**: doi:10.1177/1971400916688354

25. Wang S, Martinez-Lage M, Sakai Y, Chawla S, Kim SG, Alonso-Basanta M, Lustig RA, Brem S, Mohan S, Wolf RL, et al. Differentiating Tumor Progression from Pseudoprogression in Patients with Glioblastomas Using Diffusion Tensor Imaging and Dynamic Susceptibility Contrast MRI. *Am J Neuroradiol* (2016) **37**: doi:10.3174/ajnr.A4474

26. Prager AJ, Martinez N, Beal K, Omuro A, Zhang Z, Young RJ. Diffusion and Perfusion MRI to Differentiate Treatment-Related Changes Including Pseudoprogression from Recurrent Tumors in High-Grade Gliomas with Histopathologic Evidence. *Am J Neuroradiol* (2015) **36**: doi:10.3174/ajnr.A4218

27. Cha J, Kim ST, Kim H-J, Kim B -j., Kim YK, Lee JY, Jeon P, Kim KH, Kong D -s., Nam D-H. Differentiation of Tumor Progression from Pseudoprogression in Patients with Posttreatment Glioblastoma Using Multiparametric Histogram Analysis. *Am J Neuroradiol* (2014) **35**: doi:10.3174/ajnr.A3876

28. Di Costanzo A, Scarabino T, Trojsi F, Popolizio T, Bonavita S, de Cristofaro M, Conforti R, Cristofano A, Colonnese C, Salvolini U, et al. Recurrent glioblastoma multiforme versus radiation injury: a multiparametric 3-T MR approach. *Radiol Med* (2014) **119**: doi:10.1007/s11547-013-0371-y

29. Seeger A, Braun C, Skardelly M, Paulsen F, Schittenhelm J, Ernemann U, Bisdas S. Comparison of Three Different MR Perfusion Techniques and MR Spectroscopy for Multiparametric Assessment in Distinguishing Recurrent High-Grade Gliomas from Stable Disease. *Acad Radiol* (2013) **20**: doi:10.1016/j.acra.2013.09.003

30. Matsusue E, Fink JR, Rockhill JK, Ogawa T, Maravilla KR. Distinction between glioma progression and post-radiation change by combined physiologic MR imaging. *Neuroradiology* (2010) **52**: doi:10.1007/s00234-009-0613-9

31. Choi YJ, Kim HS, Jahng GH, Kim SJ, Suh DC. Pseudoprogression in patients with glioblastoma: Added value of arterial spin labeling to dynamic susceptibility contrast perfusion MR imaging. *Acta radiol* (2013) **54**:448–454. doi:10.1177/0284185112474916

32. Park JE, Kim HS, Goh MJ, Kim SJ, Kim JH. Pseudoprogression in patients with glioblastoma: Assessment by using volume-weighted voxel-based multiparametric clustering of MR imaging data in an independent test set. *Radiology* (2015) **275**: doi:10.1148/radiol.14141414

33. Dillman D, Smyth J, Christian L. *Internet, phone,mail, and mixed-mode surveys : the tailored design method.* 4th ed. Hoboken: Wiley (2014).
